# Supplementary material for: Energy Landscapes and Heat Capacity Signatures for Monomers and Dimers of Amyloid-Forming Hexapeptides
Source: Int J Mol Sci. 2023 Jun 25;24(13):10613. doi: 10.3390/ijms241310613 (PMC10341876; doi:10.3390/ijms241310613)
Supplement: Supplementary file 1 [file ijms-24-10613-s001.zip › ijms-2432437-supplementary.pdf]

Energy landscapes and heat capacity signatures for monomers and dimers of amyloid forming hexapeptides

Nicy and David J. Wales *

Yusuf Hamied Department of Chemistry, University of Cambridge, Cambridge, CB2 1EW, UK

* Correspondence: dw34@cam.ac.uk

(Dated: 16 June 2023)

SUPPLEMENTARY INFORMATION

- Table S1 contains the details of sequence numbers for the hexapeptides.
- Tables S2, S3, and S4 contain the details of temperatures at which features are observed in the C_V.
- Figure S1 shows correlation plots between different amyloid predictors.
- Figures S2–S31 show disconnectivity graphs for all the hexapeptides.

UniProt Identifier	Hexapeptide	Sequence
P10997 IAPP HUMAN	NFGAIL	55−60
	GAILSS	57−62
	SSTNVG	61-66
P12969 IAPP RAT	NLGPVL	59−64
P10636 TAU HUMAN	VQIVYK	623−628
P01308 INS HUMAN	LYQLEN	102−107
	YQLENY	103−108
P61769 B2MG HUMAN	LLYYTE	84−89
	YYTEFT	86−91
	EVDLLK	56−61
	LSFSKD	74−79
	NGERIE	62−67
P05067 A4 HUMAN	KLVFFA	687−692
	GAIIGL	700−705
	MVGGVV	706−711
	GGVVIA	708−713
P02649 APOE HUMAN	SSQVTQ	71−76
P04156 PRIO HUMAN	SNQNNF	170−175
P02743 SAMP HUMAN	GYVIIK	213−218

Table S1: Sequence numbers of hexapeptides in their respective proteins as obtained from the UniProt database.

Hexapeptide	Peak/Inflection(*) temperatures			
	Feature 1	Feature 2	Feature 3	Feature 4
**NFGAIL**	0.076*	0.364*	0.701	-
NAGAIL	0.167*	0.524	-	-
LIAGFN	0.043	0.223*	0.392	0.962
NLGPVL	0.055	0.456	-	-
**STVIIE**	0.248	0.564	-	-
STVIIP	0.012	0.157	0.390	0.945
SPVIIE	0.030	0.295	-	-
STVVIE	0.101	0.235	0.947	-
**VQIVYK**	0.089	0.518	0.891	-
VQIVEK	0.011	0.131*	0.269	1.031
NAEVYK	0.047	0.216	0.898	-
**LYQLEN**	0.079*	0.227	0.385	0.802
YQLENY	0.024	0.281	-	-
**LLYYTE**	0.041	0.178	0.316	-
YYTEFT	0.017	0.242	0.427*	1.145

Table S2: Hexapeptides, and $k_{\mathrm{B}}T$ (kcal mol^{-1}) at which peaks or distinct inflection points are observed. The features are labelled as 1, 2, 3, and 4 from low temperature to high temperature. The inflection points are marked with an asterisk (*).

Hexapeptide	Peak/Inflection(*) temperatures			
	Feature 1	Feature 2	Feature 3	Feature 4
GAIIGL	0.219*	0.598	-	-
GGVVIA	0.034	0.377*	0.582	-
MVGGVV	0.037	0.213	0.630	-
GAILSS	0.179	0.310	-	-
SSQVTQ	0.218	0.426	-	-
SSTNVG	0.054	0.241	-	-
SNQNNF	0.016	0.199	0.312	1.011
SVSSSY	0.039	0.240	0.514	-
GYVIIK	0.016	0.437	-	-
KLVFFA	0.150	0.439	-	-
KAFIIQ	0.259	-	-	-
KAILFL	0.112	0.309	-	-
EVDLLK	0.021	0.204*	0.417	-
LSFSKD	0.011	0.101*	0.368	0.860
NGERIE	0.155	0.404	-	-

Table S3: Hexapeptides, and $k_{\mathrm{B}}T$ (kcal mol^{-1}) at which peaks or distinct inflection points are observed. The features are labelled as 1, 2, 3, and 4 from low temperature to high temperature. The inflection points are marked with an asterisk (*).

Hexapeptide	Peak/Inflection(*) temperatures			
	Feature 1	Feature 2	Feature 3	Feature 4
**NFGAIL**	0.060	0.432*	0.859	-
NAGAIL	0.016	0.107	1.148	-
LIAGFN	0.167	0.294*	1.080	-
NLGPVL	0.100	0.458^{i}	1.095	-
**STVIIE**	0.122	0.540	1.096^{i}	-
STVIIP	0.036	0.547	1.051	-
SPVIIE	0.023	0.229	0.400	-
STVVIE	0.043	0.354	0.667	-
**VQIVYK**	0.081*	0.268	1.123	-
VQIVEK	0.061	0.420	0.891	-
NAEVYK	0.107	0.718	-	-
**LYQLEN**	0.042	0.173*	0.600	0.888
YQLENY	0.271*	0.408	-	-
**LLYYTE**	0.021^{i}	0.281	0.902	-
YYTEFT	0.057	0.215	0.418	1.156^{i}

Table S4: Dimers of hexapeptides, and $k_{\mathrm{B}}T$ (kcal mol^{-1}) at which peaks or distinct inflection points are observed. The features are labelled as 1, 2, 3, and 4 from low temperature to high temperature. The inflection points are marked with an asterisk (*) and very small peaks are marked with 'i' (^{i}).

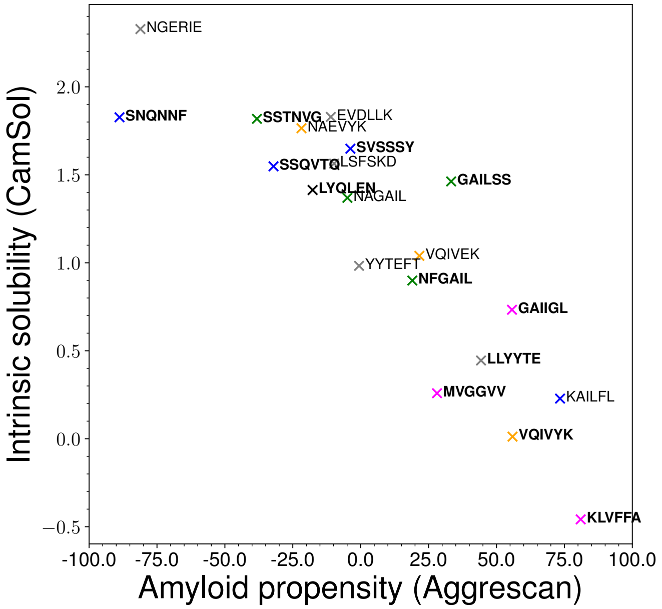

(a) Correlation plot between amyloid predictor Aggrescan and intrinsic solubility predictor CamSol.

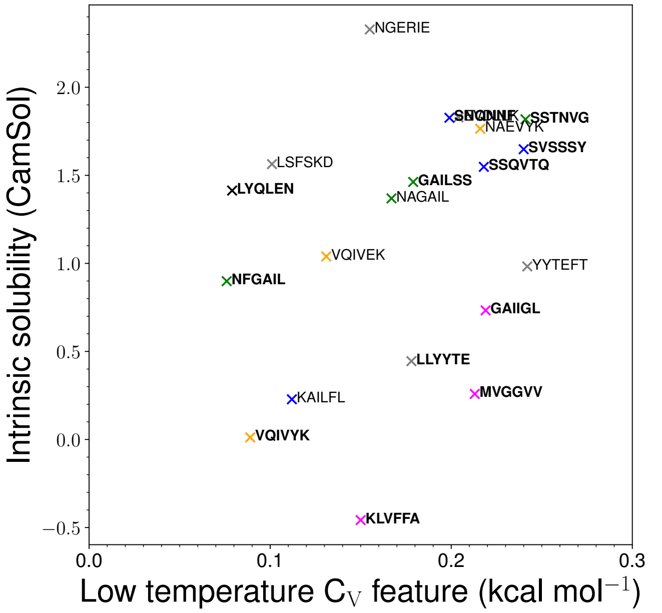

(b) Correlation plot between low temperature heat capacity feature and intrinsic solubility as predicted using CamSol.

Figure S1: Correlation plots between different amyloid predictors.

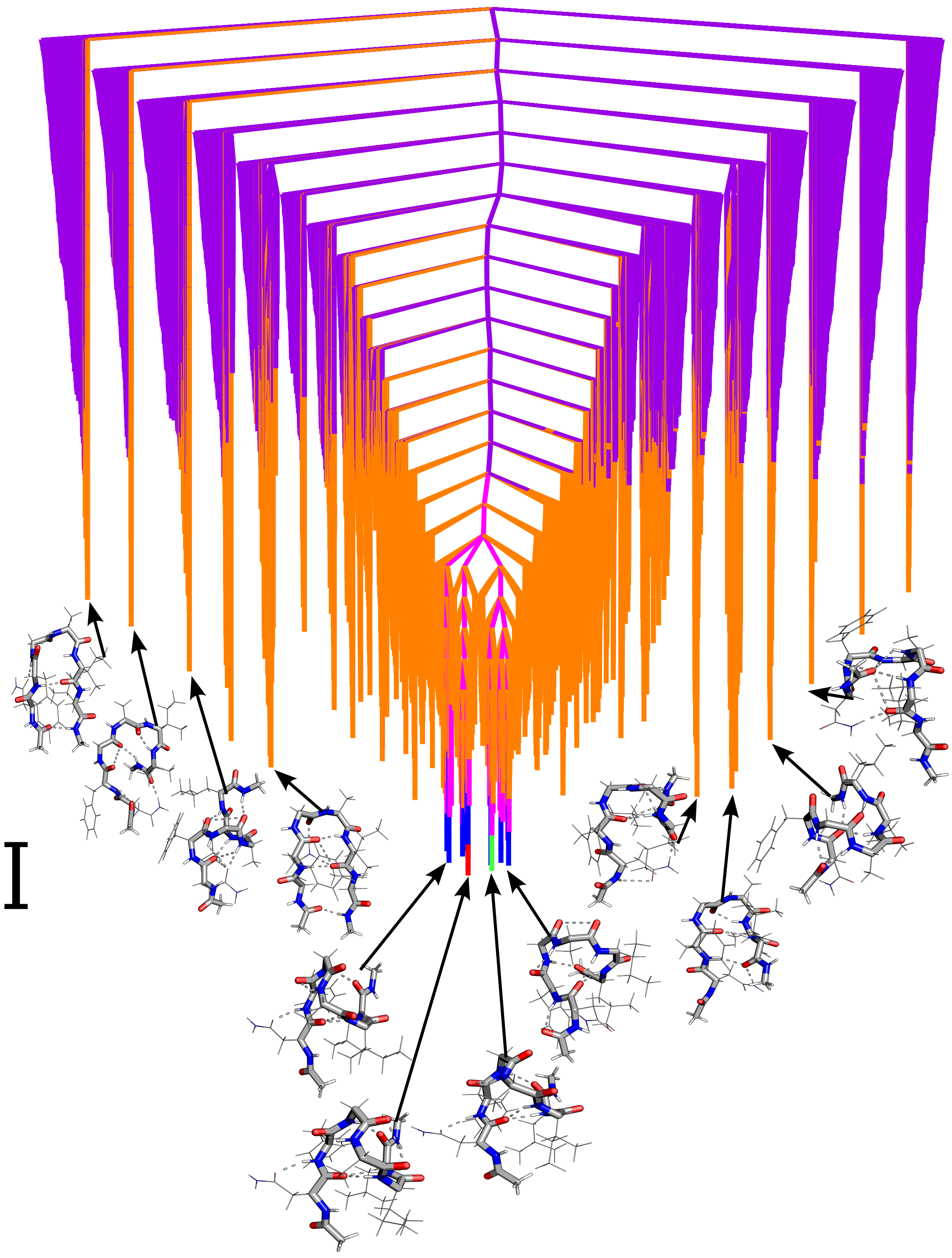

Figure S2: Disconnectivity graphs for **NFGAIL** monomer. Local minima representing transition for peaks/inflection points are represented by red to blue (peak 1), green to orange (peak 2), pink to purple (peak 3) and grey to yellow (peak 4). The scalebar represents 1 kcal mol^{-1}.

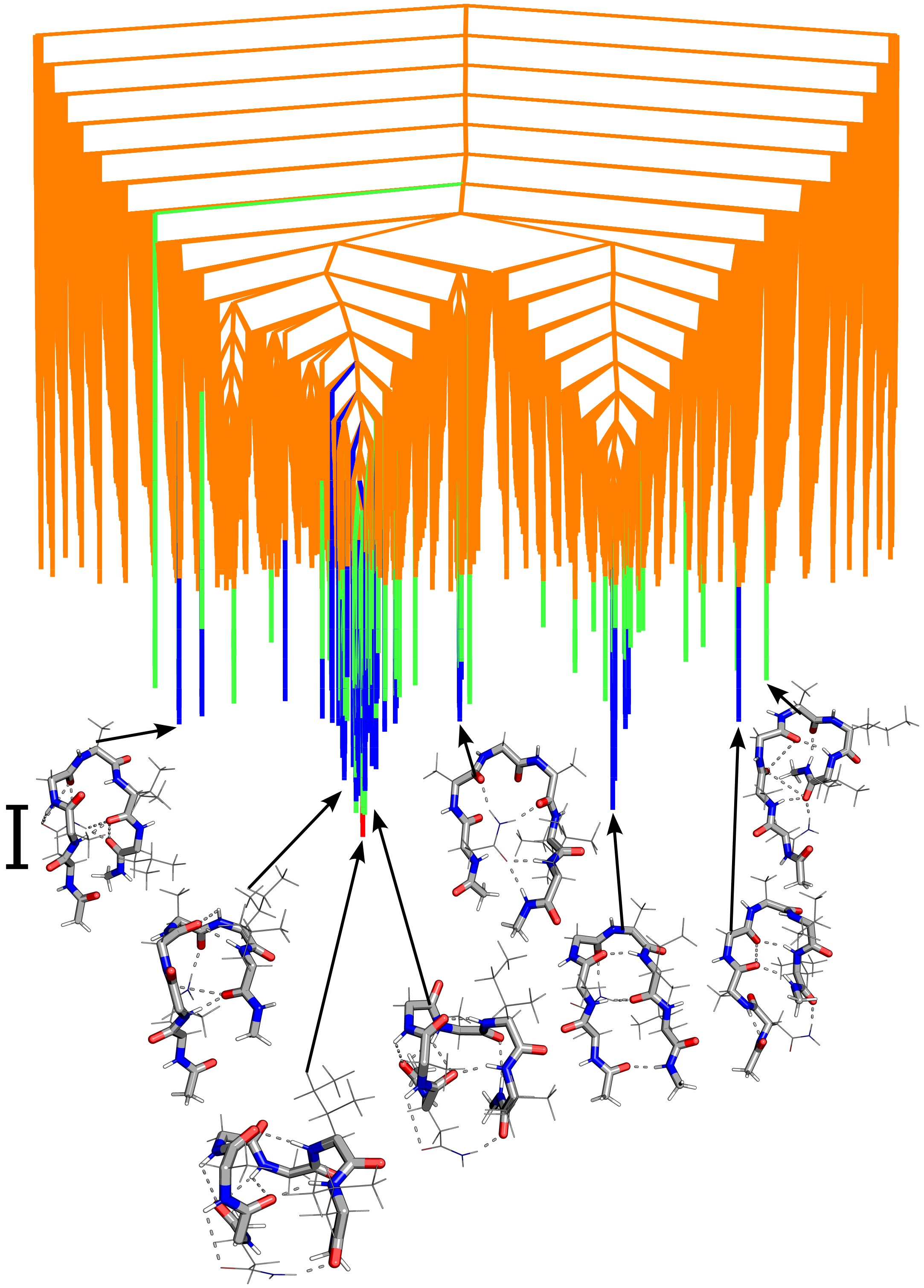

Figure S3: Disconnectivity graphs for NAGAIL monomer. Local minima representing transition for peaks/inflection points are represented by red to blue (peak 1), green to orange (peak 2), pink to purple (peak 3) and grey to yellow (peak 4). The scalebar represents 1 kcal mol^{-1}.

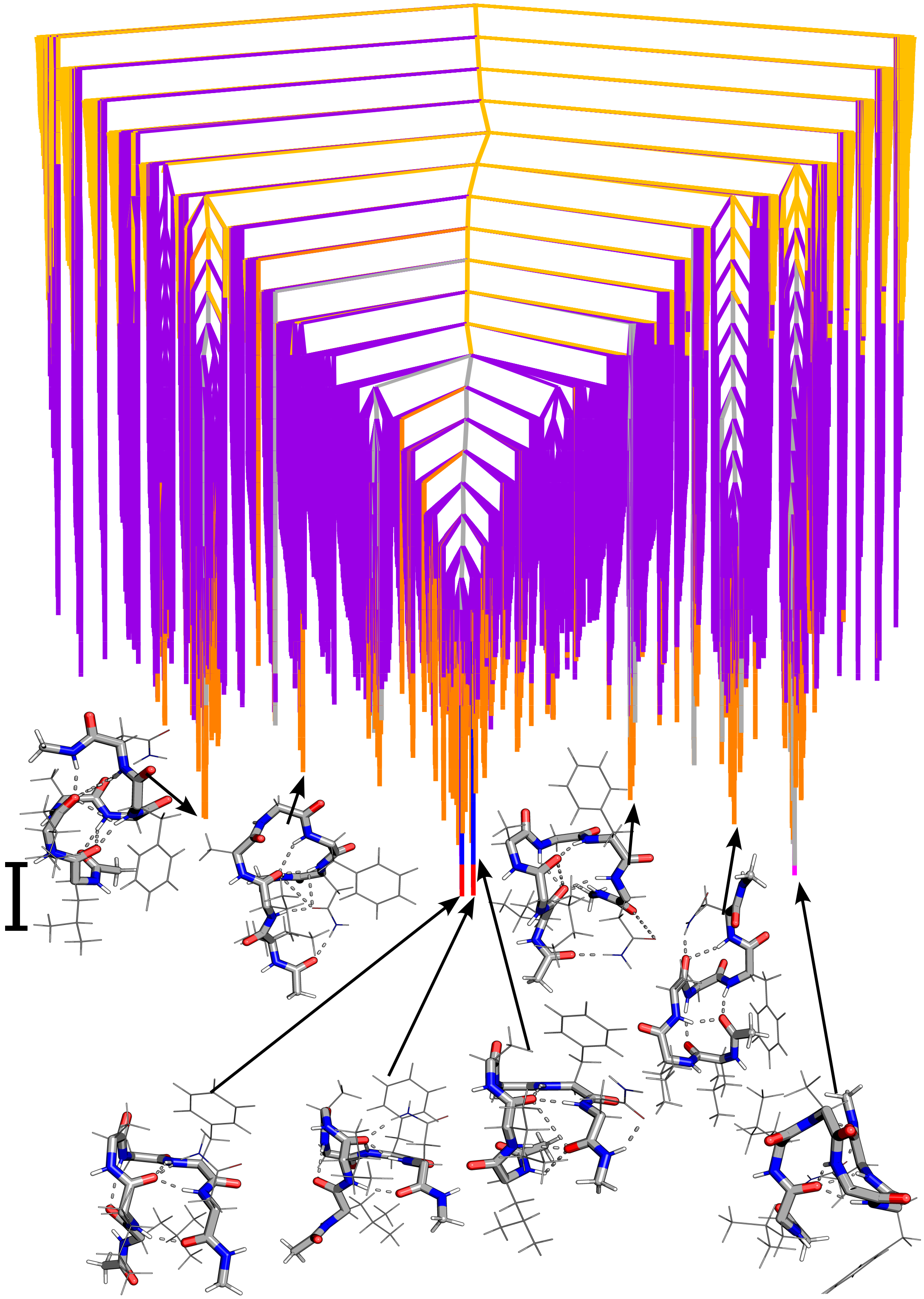

Figure S4: Disconnectivity graphs for LIAGFN monomer. Local minima representing transition for peaks/inflection points are represented by red to blue (peak 1), green to orange (peak 2), pink to purple (peak 3) and grey to yellow (peak 4). The scalebar represents 1 kcal mol^{-1}.

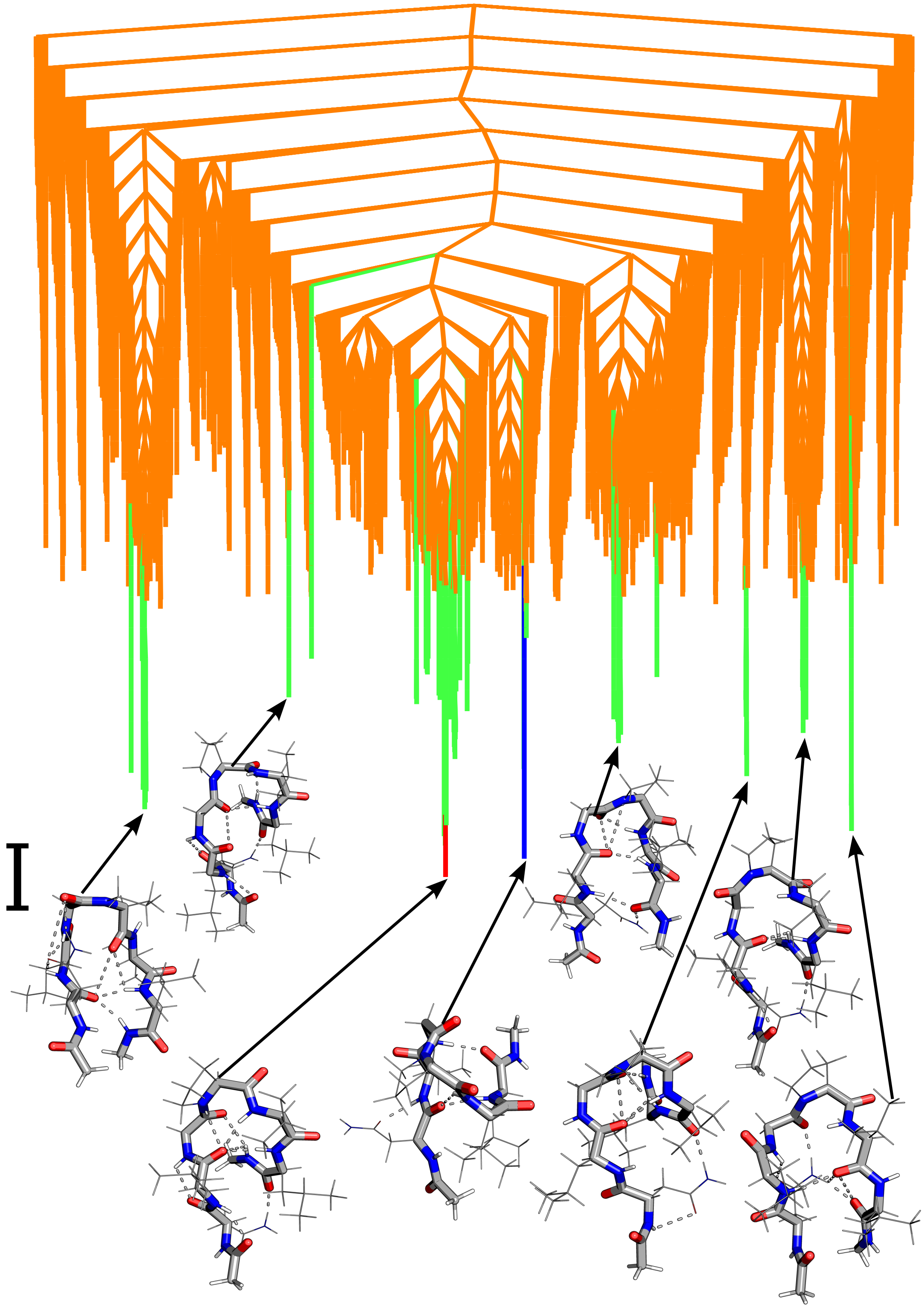

Figure S5: Disconnectivity graphs for NLGPVL monomer. Local minima representing transition for peaks/inflection points are represented by red to blue (peak 1), green to orange (peak 2), pink to purple (peak 3) and grey to yellow (peak 4). The scalebar represents 1 kcal mol^{-1}.

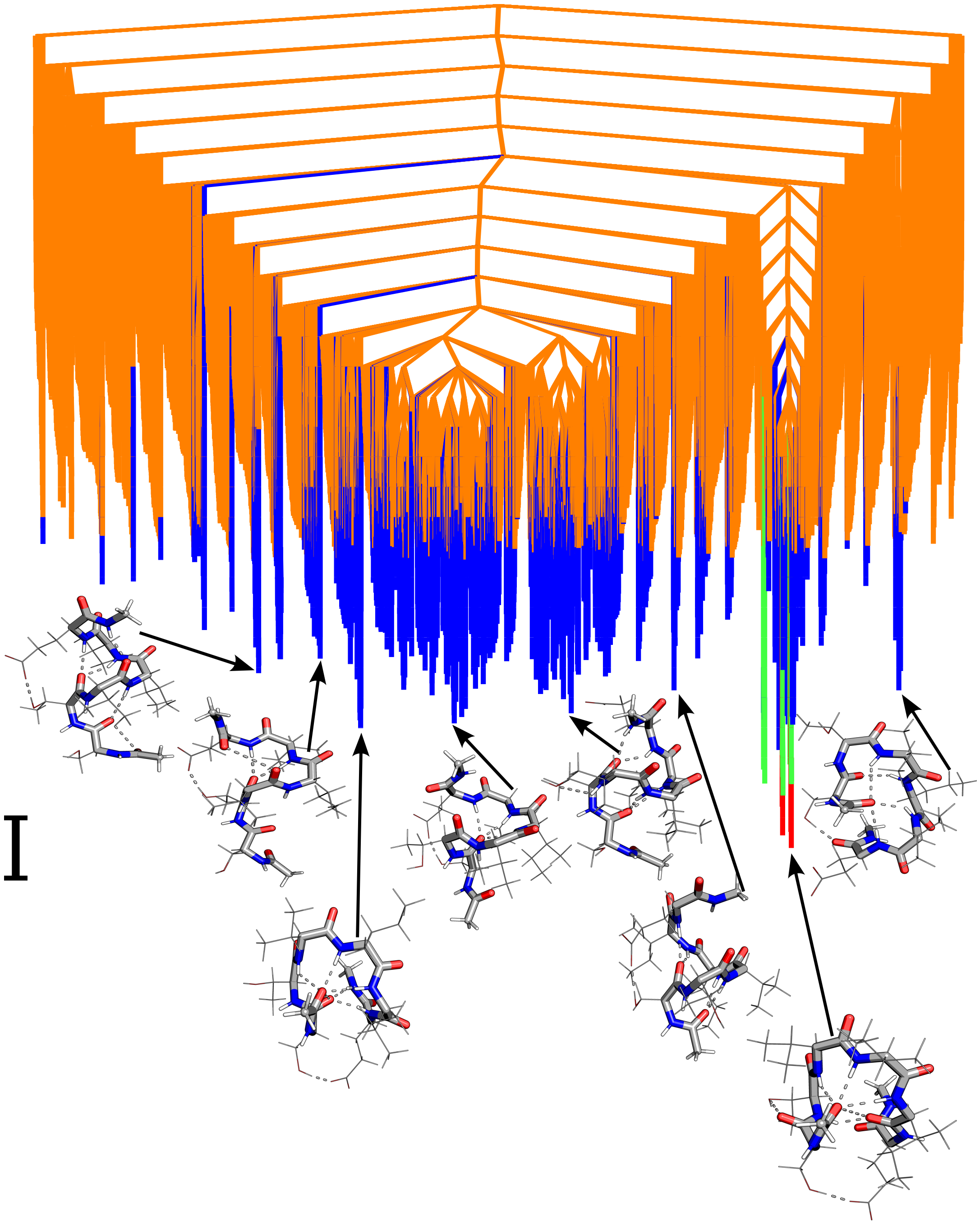

Figure S6: Disconnectivity graphs for **STVIIE** monomer. Local minima representing transition for peaks/inflection points are represented by red to blue (peak 1), green to orange (peak 2), pink to purple (peak 3) and grey to yellow (peak 4). The scalebar represents 1 kcal mol^{-1}.

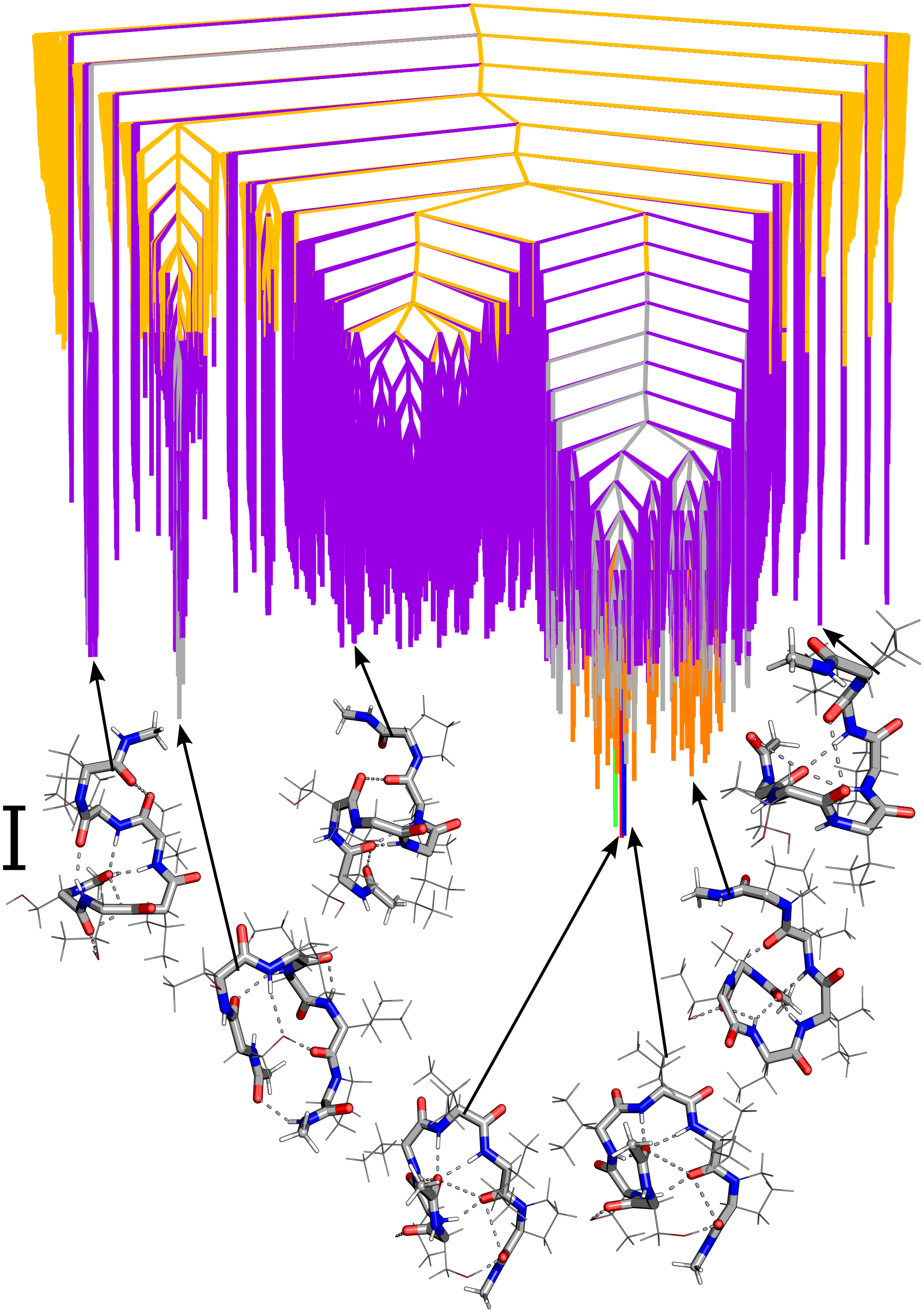

Figure S7: Disconnectivity graphs for STVIIP monomer. Local minima representing transition for peaks/inflection points are represented by red to blue (peak 1), green to orange (peak 2), pink to purple (peak 3) and grey to yellow (peak 4). The scalebar represents 1 kcal mol^{-1}.

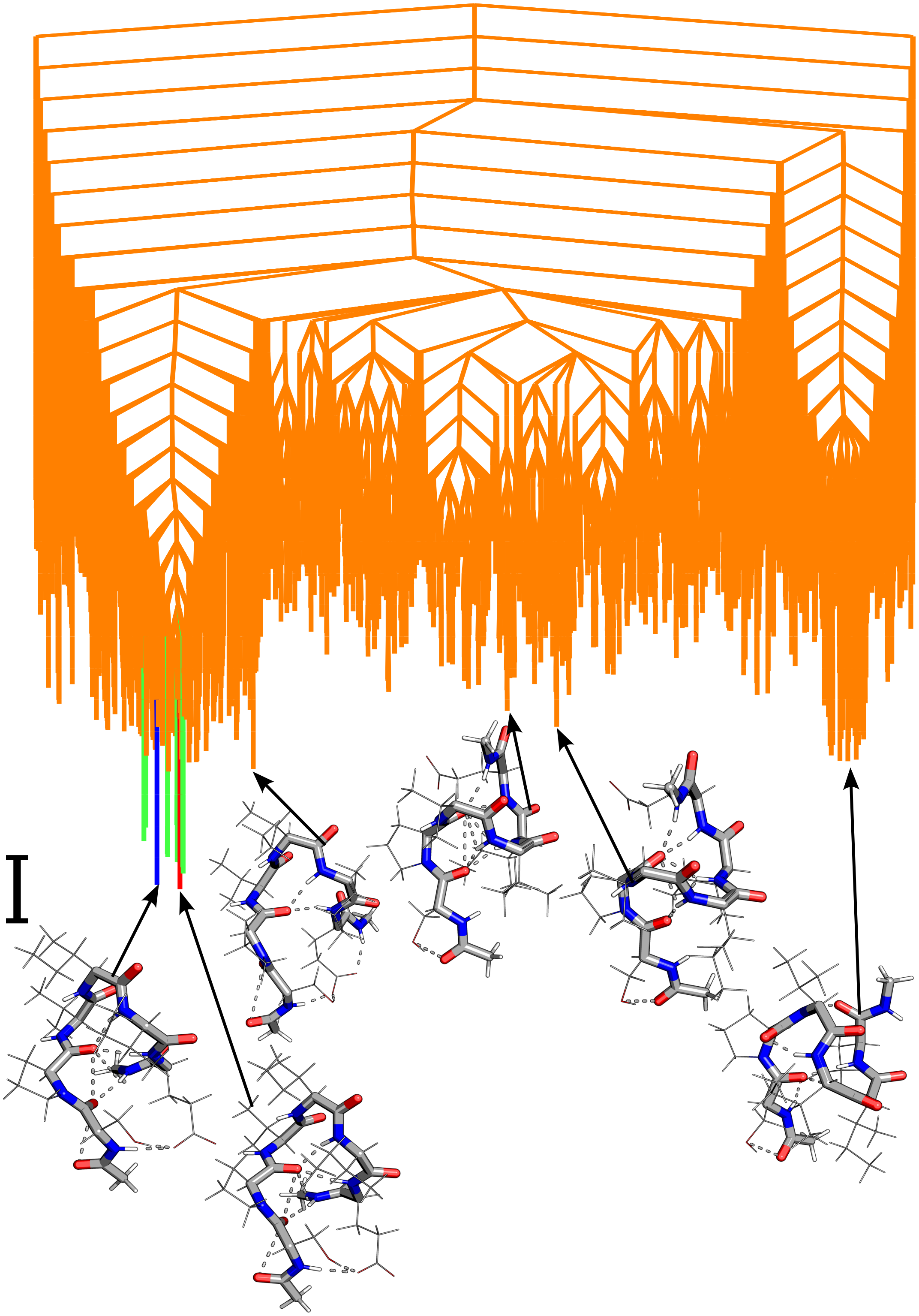

Figure S8: Disconnectivity graphs for SPVIIE monomer. Local minima representing transition for peaks/inflection points are represented by red to blue (peak 1), green to orange (peak 2), pink to purple (peak 3) and grey to yellow (peak 4). The scalebar represents 1 kcal mol^{-1}.

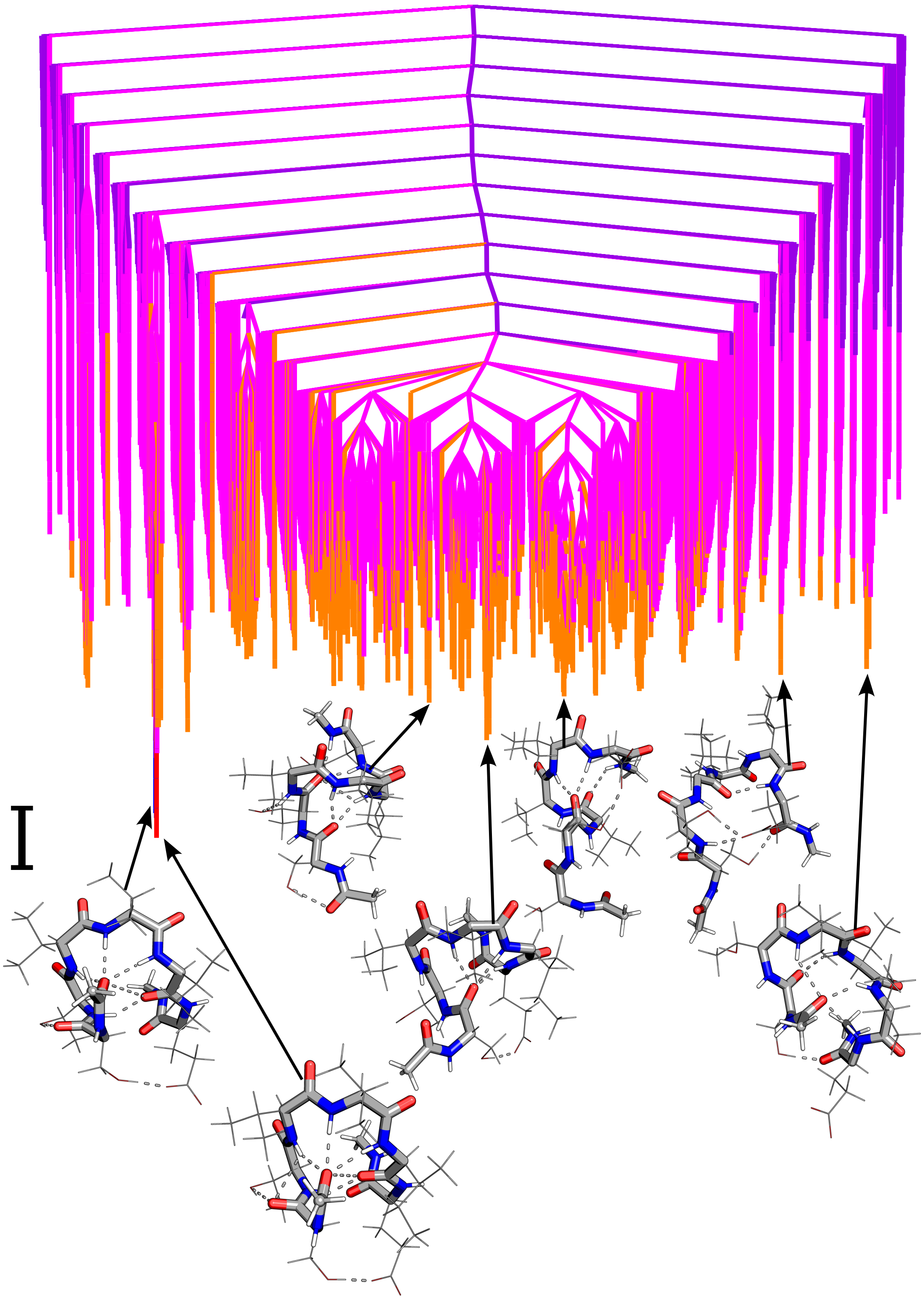

Figure S9: Disconnectivity graphs for STVVIE monomer. Local minima representing transition for peaks/inflection points are represented by red to blue (peak 1), green to orange (peak 2), pink to purple (peak 3) and grey to yellow (peak 4). The scalebar represents 1 kcal mol^{-1}.

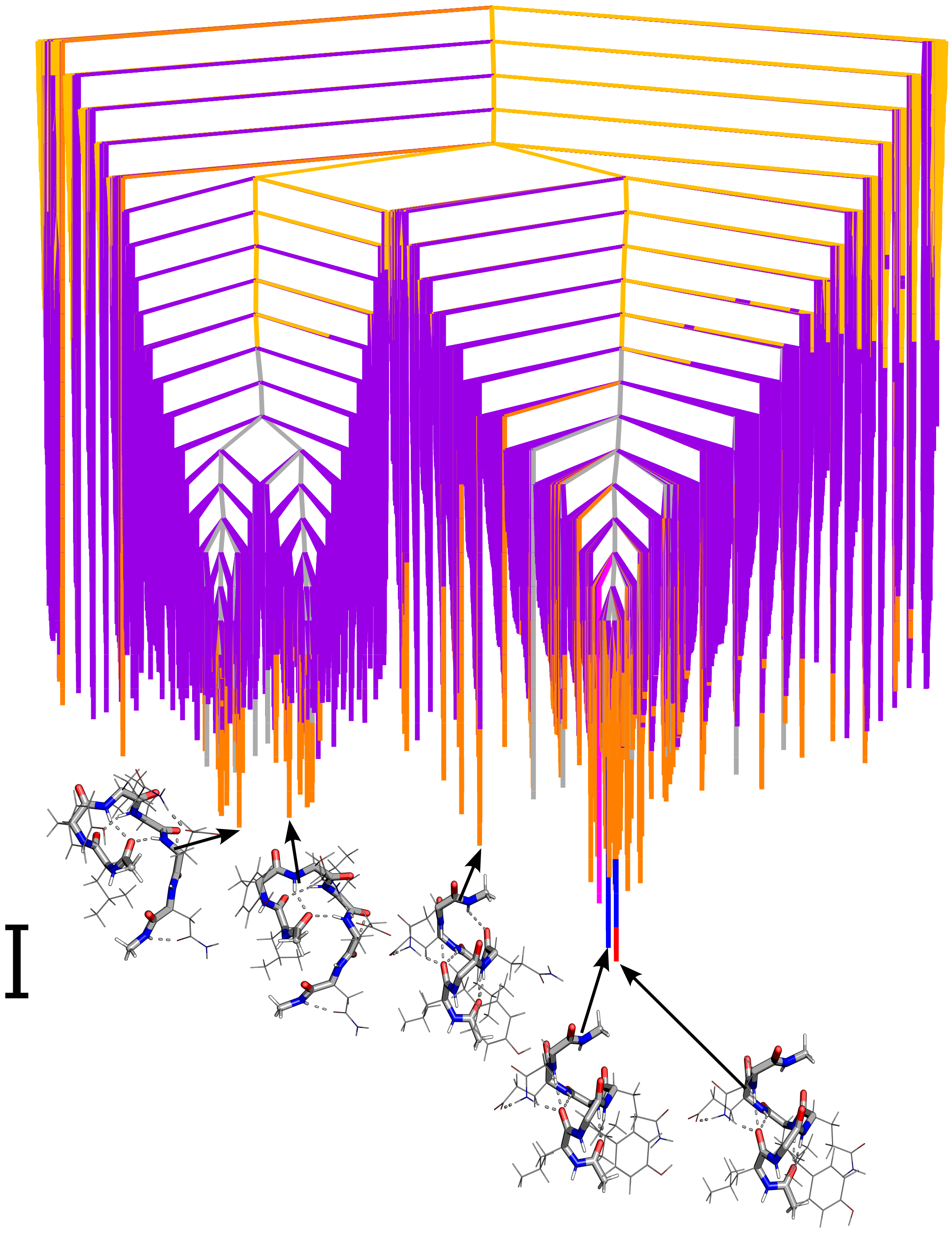

Figure S10: Disconnectivity graphs for **LYQLEN** monomer. Local minima representing transition for peaks/inflection points are represented by red to blue (peak 1), green to orange (peak 2), pink to purple (peak 3) and grey to yellow (peak 4). The scalebar represents 1 kcal mol^{-1}.

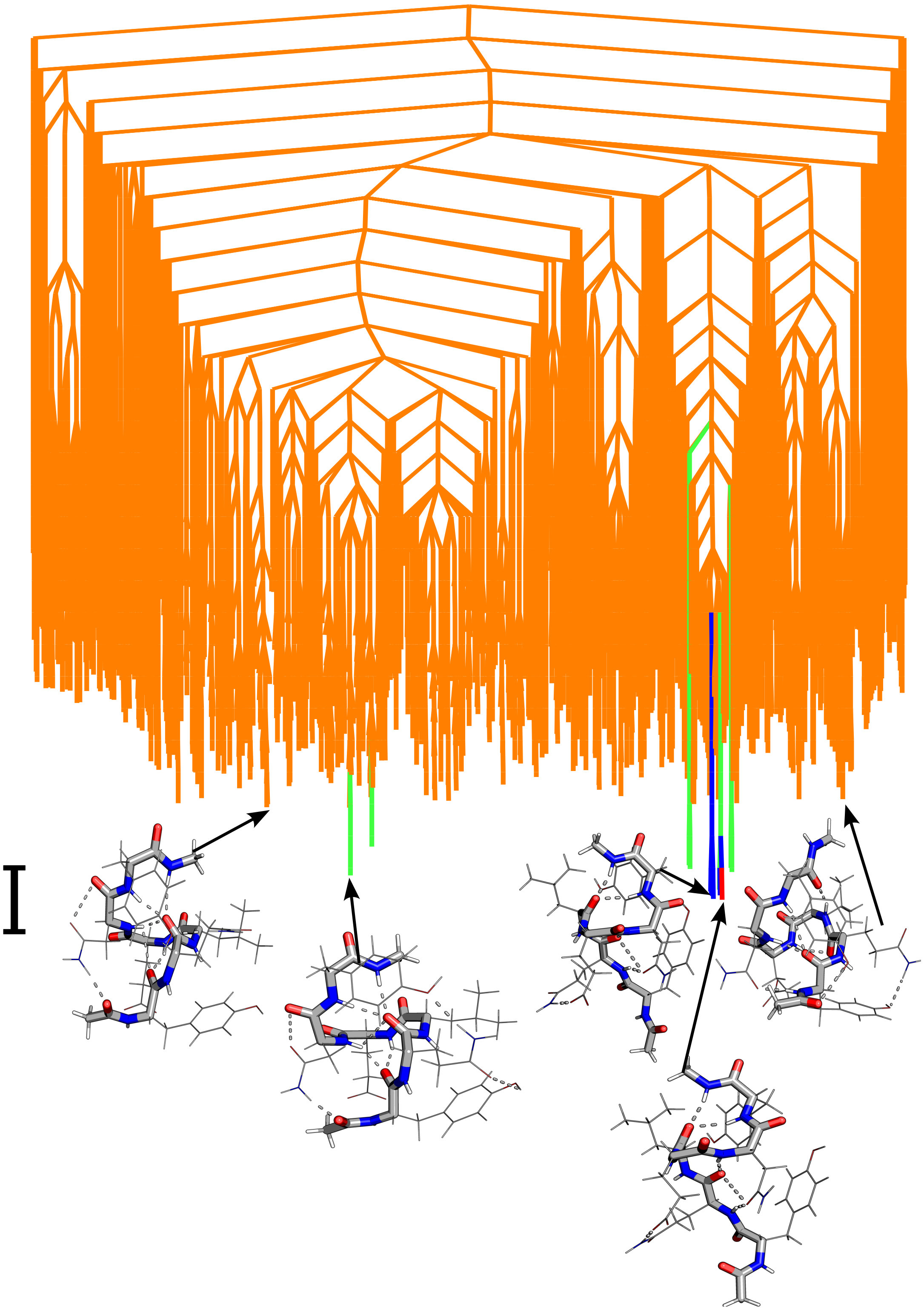

Figure S11: Disconnectivity graphs for YQLENY monomer. Local minima representing transition for peaks/inflection points are represented by red to blue (peak 1), green to orange (peak 2), pink to purple (peak 3) and grey to yellow (peak 4). The scalebar represents 1 kcal mol^{-1}.

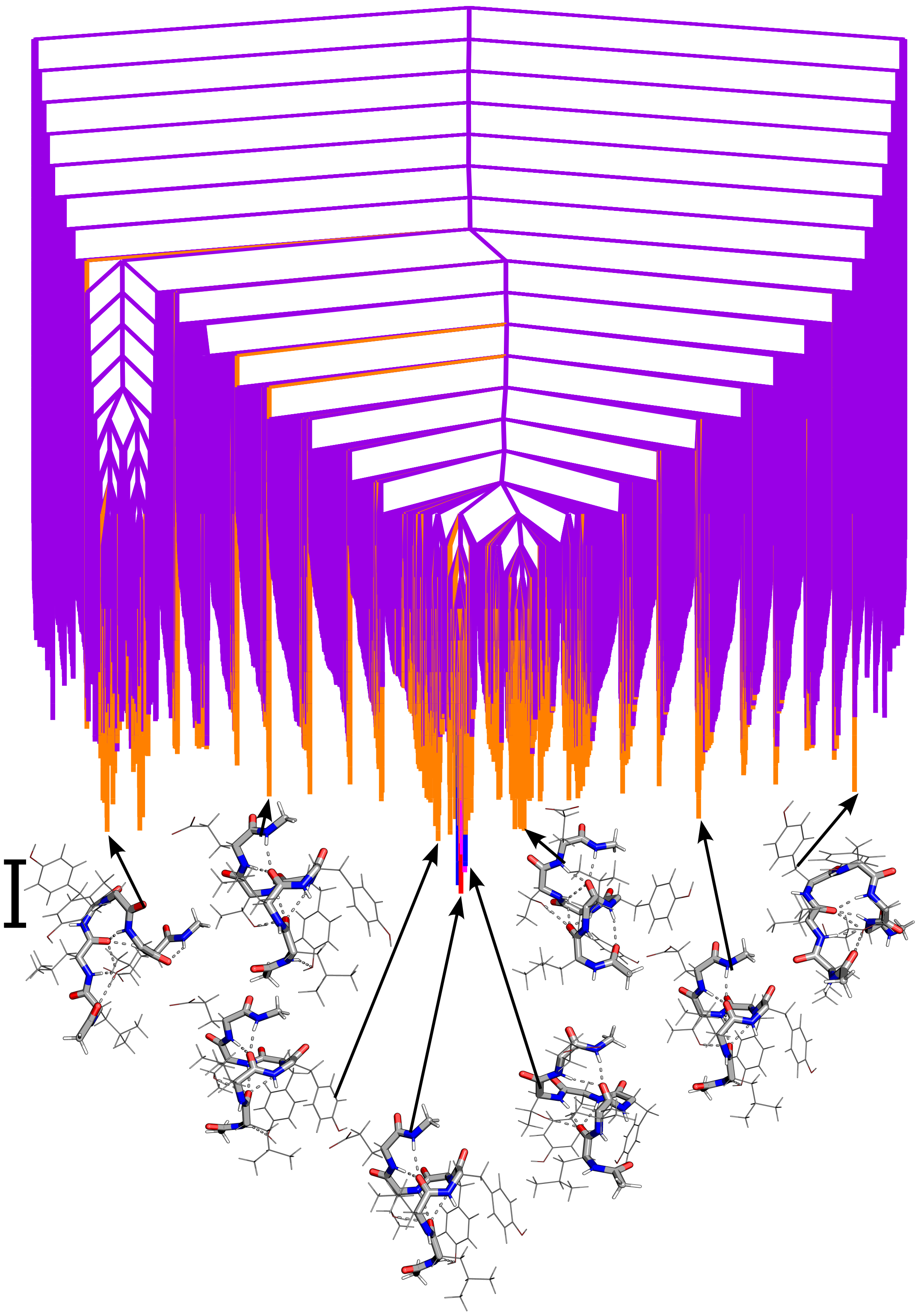

Figure S12: Disconnectivity graphs for **LLYYTE** monomer. Local minima representing transition for peaks/inflection points are represented by red to blue (peak 1), green to orange (peak 2), pink to purple (peak 3) and grey to yellow (peak 4). The scalebar represents 1 kcal mol^{-1}.

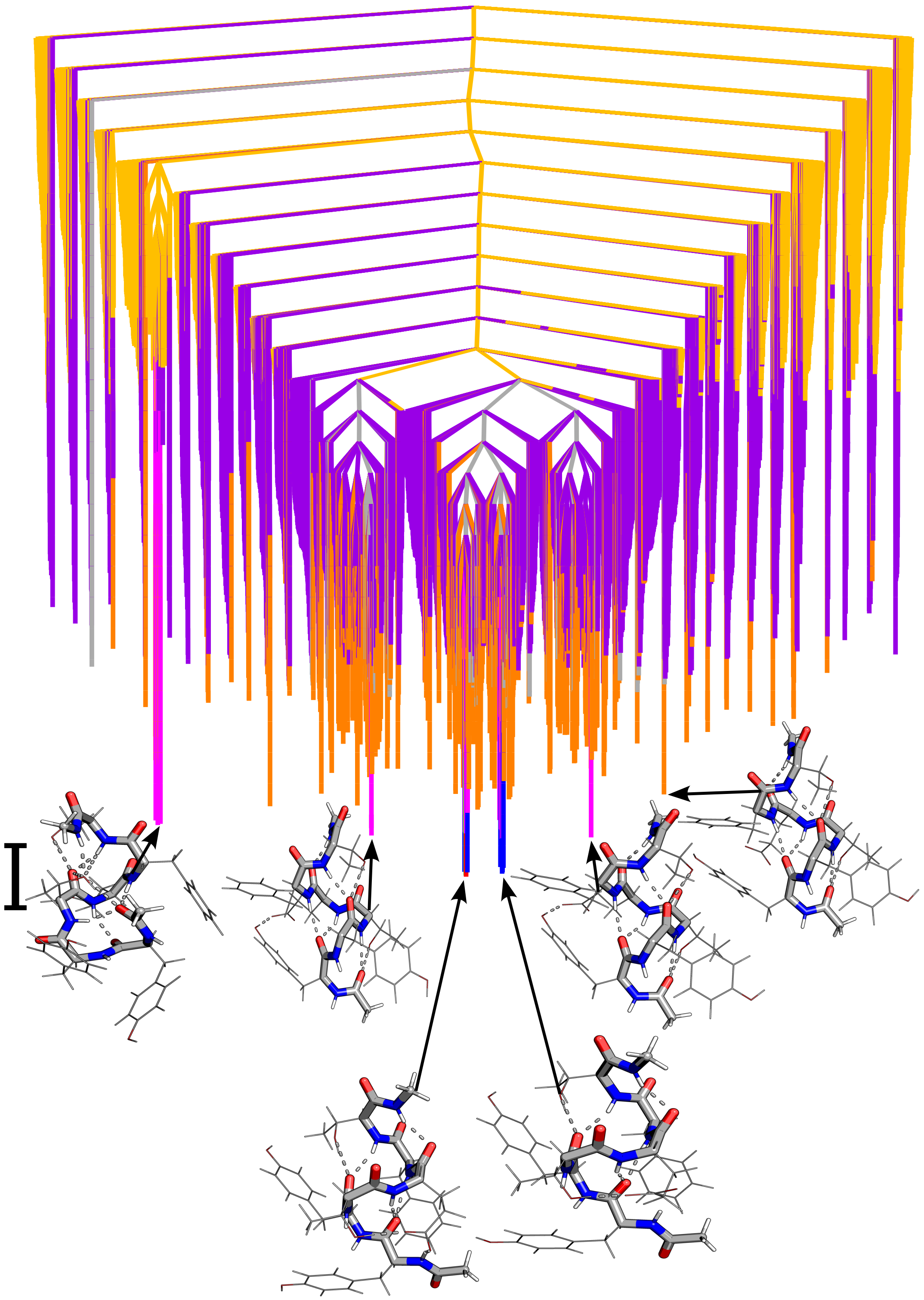

Figure S13: Disconnectivity graphs for YYTEFT monomer. Local minima representing transition for peaks/inflection points are represented by red to blue (peak 1), green to orange (peak 2), pink to purple (peak 3) and grey to yellow (peak 4). The scalebar represents 1 kcal mol^{-1}.

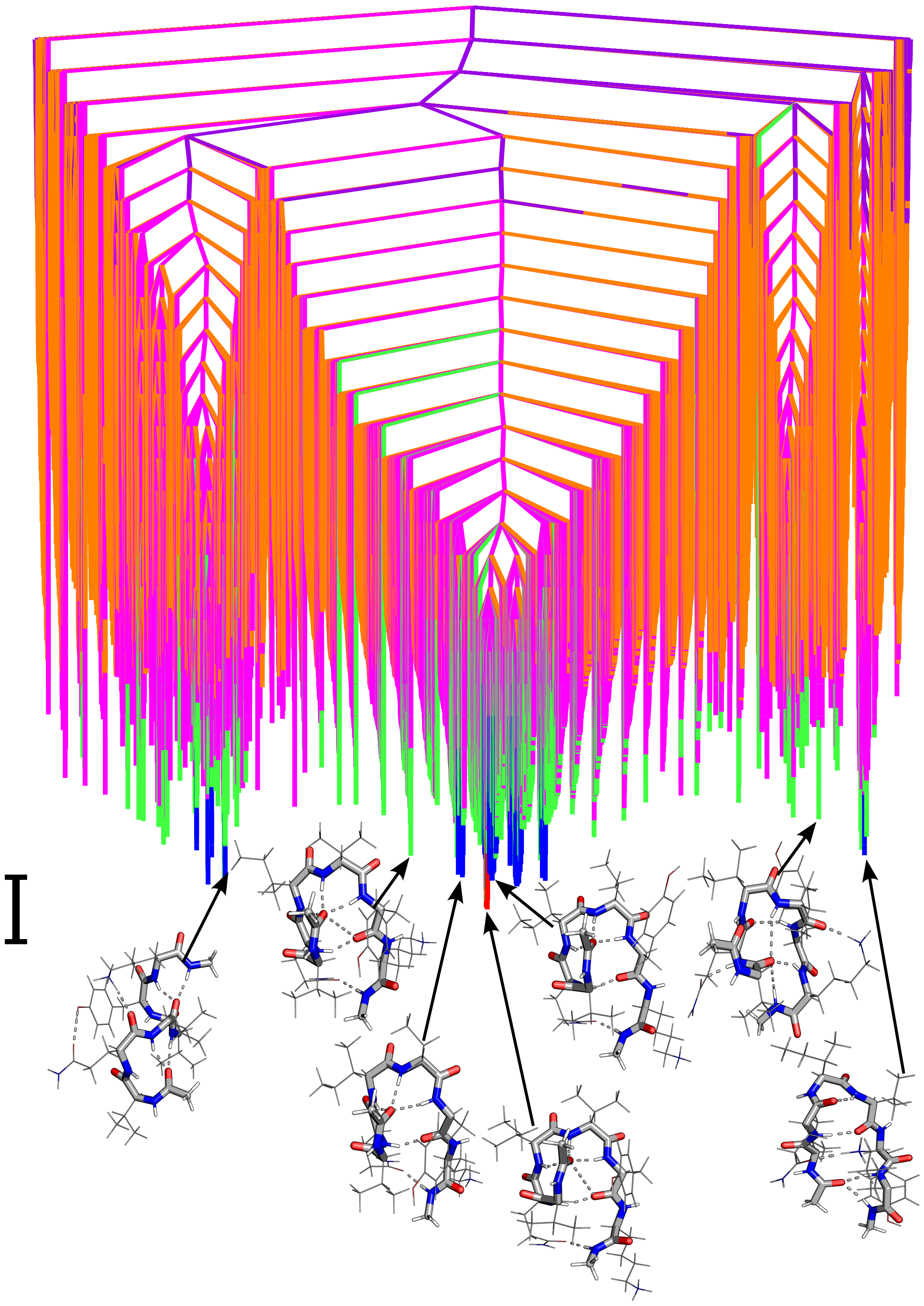

Figure S14: Disconnectivity graphs for **VQIVYK** monomer. Local minima representing transition for peaks/inflection points are represented by red to blue (peak 1), green to orange (peak 2), pink to purple (peak 3) and grey to yellow (peak 4). The scalebar represents 1 kcal mol^{-1}.

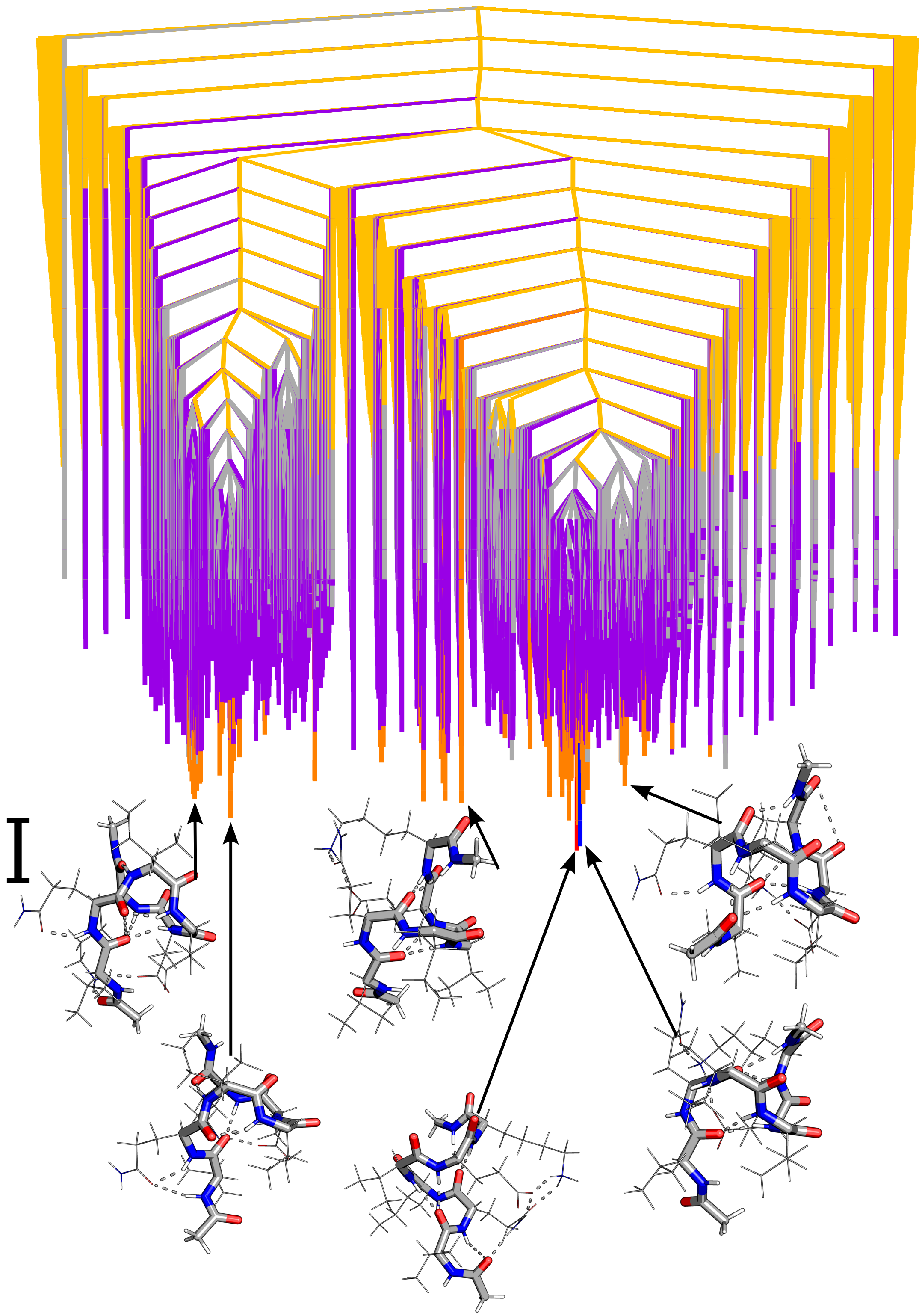

Figure S15: Disconnectivity graphs for VQIVEK monomer. Local minima representing transition for peaks/inflection points are represented by red to blue (peak 1), green to orange (peak 2), pink to purple (peak 3) and grey to yellow (peak 4). The scalebar represents 1 kcal mol^{-1}.

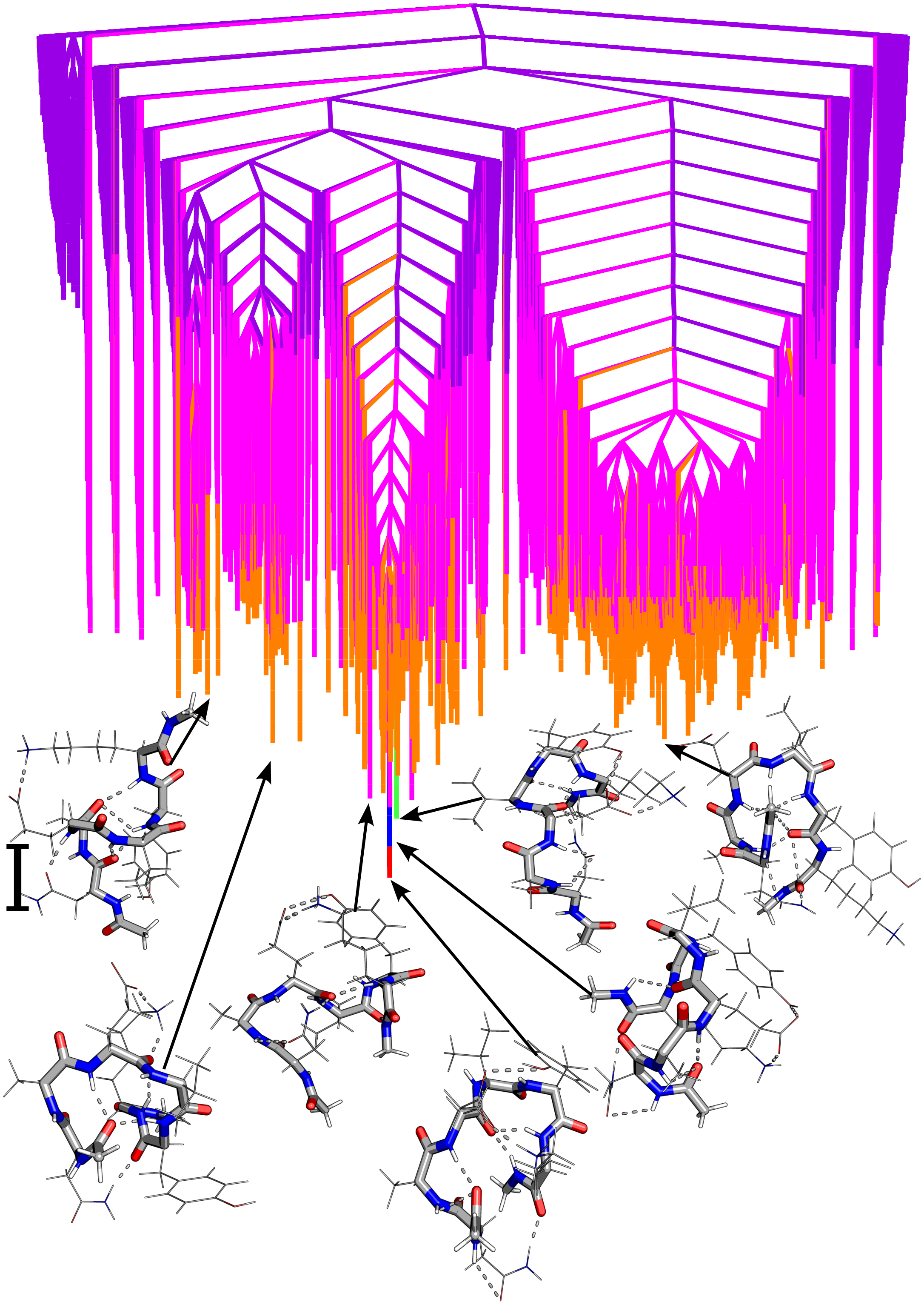

Figure S16: Disconnectivity graphs for NAEVYK monomer. Local minima representing transition for peaks/inflection points are represented by red to blue (peak 1), green to orange (peak 2), pink to purple (peak 3) and grey to yellow (peak 4). The scalebar represents 1 kcal mol^{-1}.

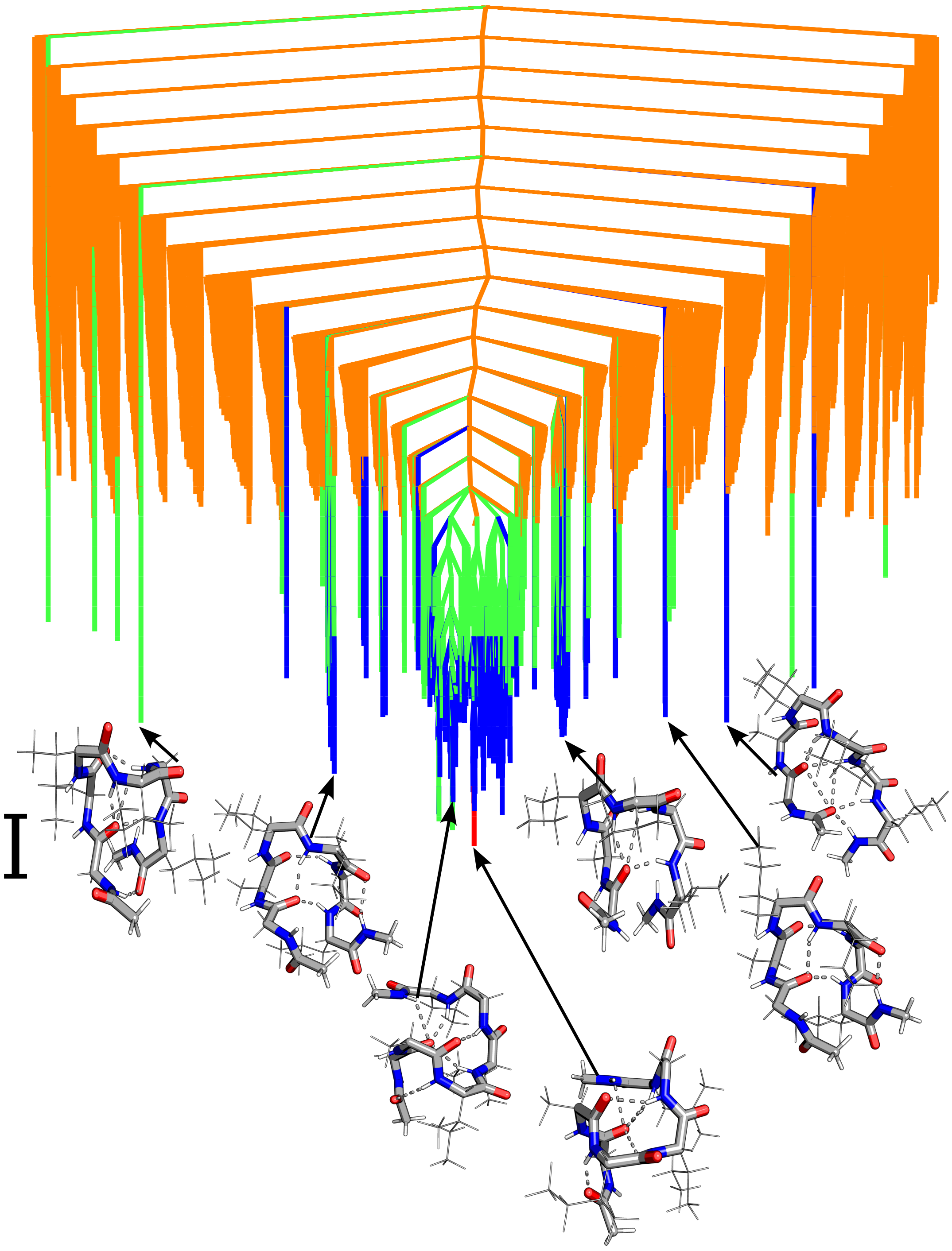

Figure S17: Disconnectivity graphs for **GAIIGL** monomer. Local minima representing transition for peaks/inflection points are represented by red to blue (peak 1), green to orange (peak 2), pink to purple (peak 3) and grey to yellow (peak 4). The scalebar represents 1 kcal mol^{-1}.

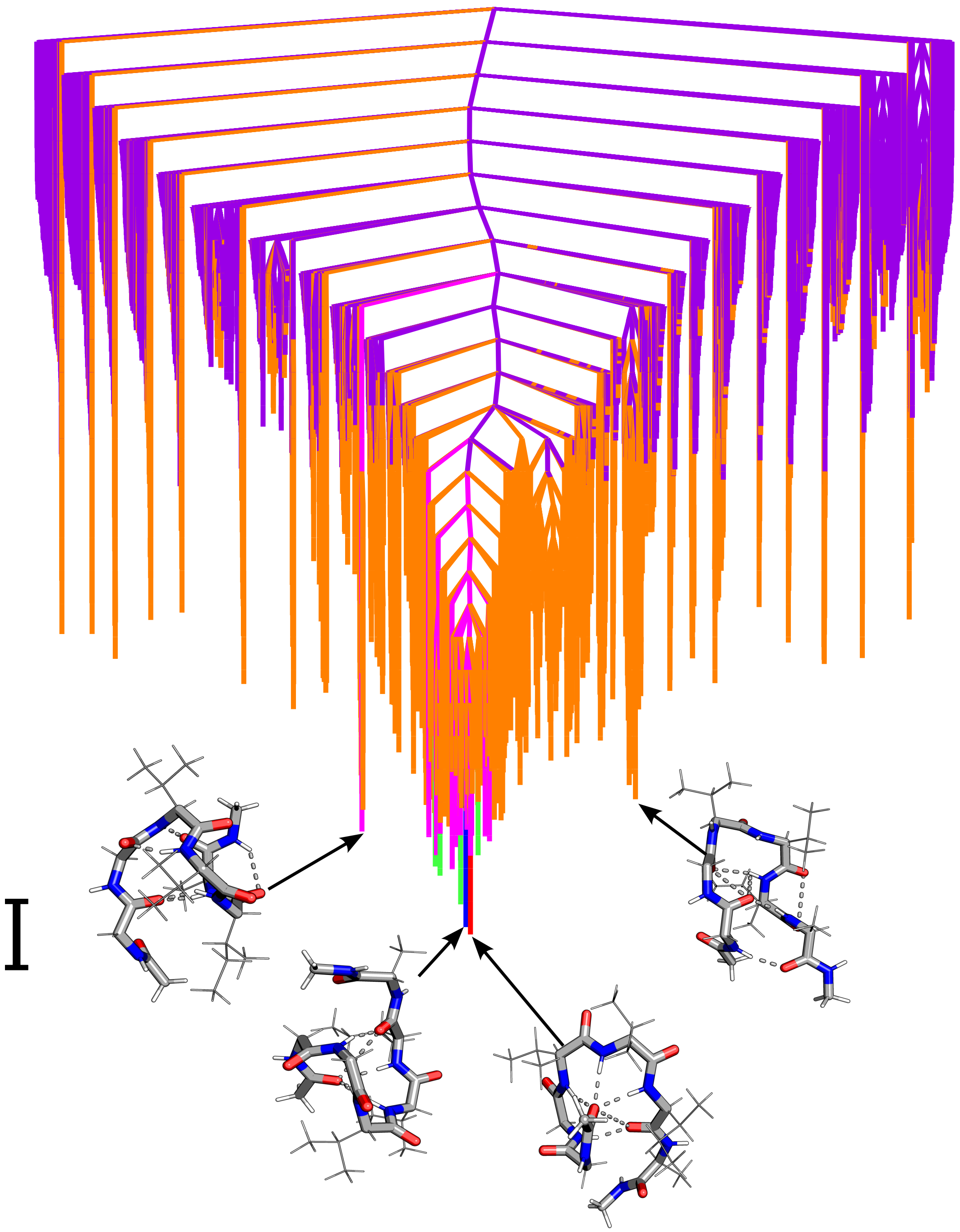

Figure S18: Disconnectivity graphs for **GGVVIA** monomer. Local minima representing transition for peaks/inflection points are represented by red to blue (peak 1), green to orange (peak 2), pink to purple (peak 3) and grey to yellow (peak 4). The scalebar represents 1 kcal mol^{-1}.

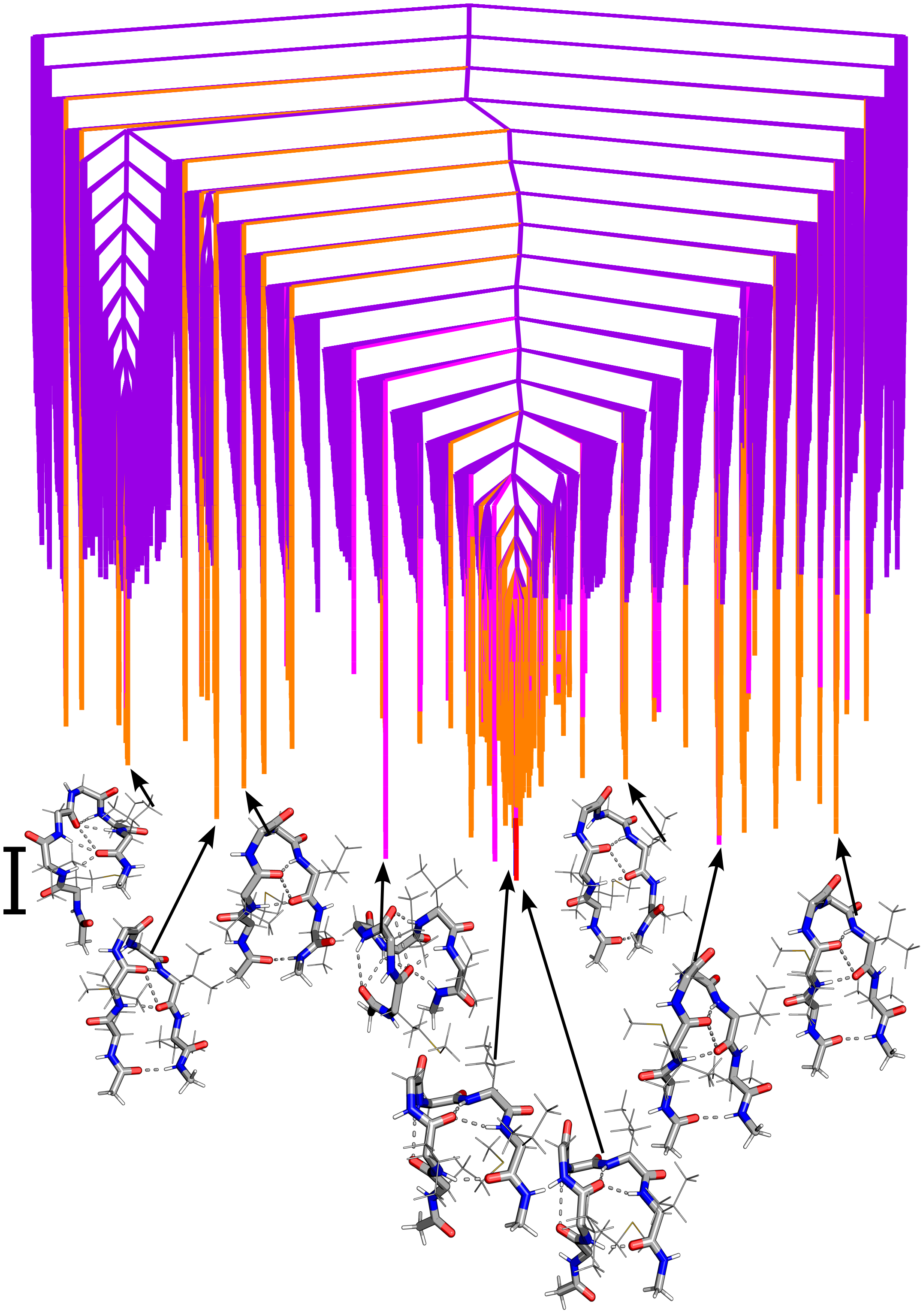

Figure S19: Disconnectivity graphs for **MVGGVV** monomer. Local minima representing transition for peaks/inflection points are represented by red to blue (peak 1), green to orange (peak 2), pink to purple (peak 3) and grey to yellow (peak 4). The scalebar represents 1 kcal mol^{-1}.

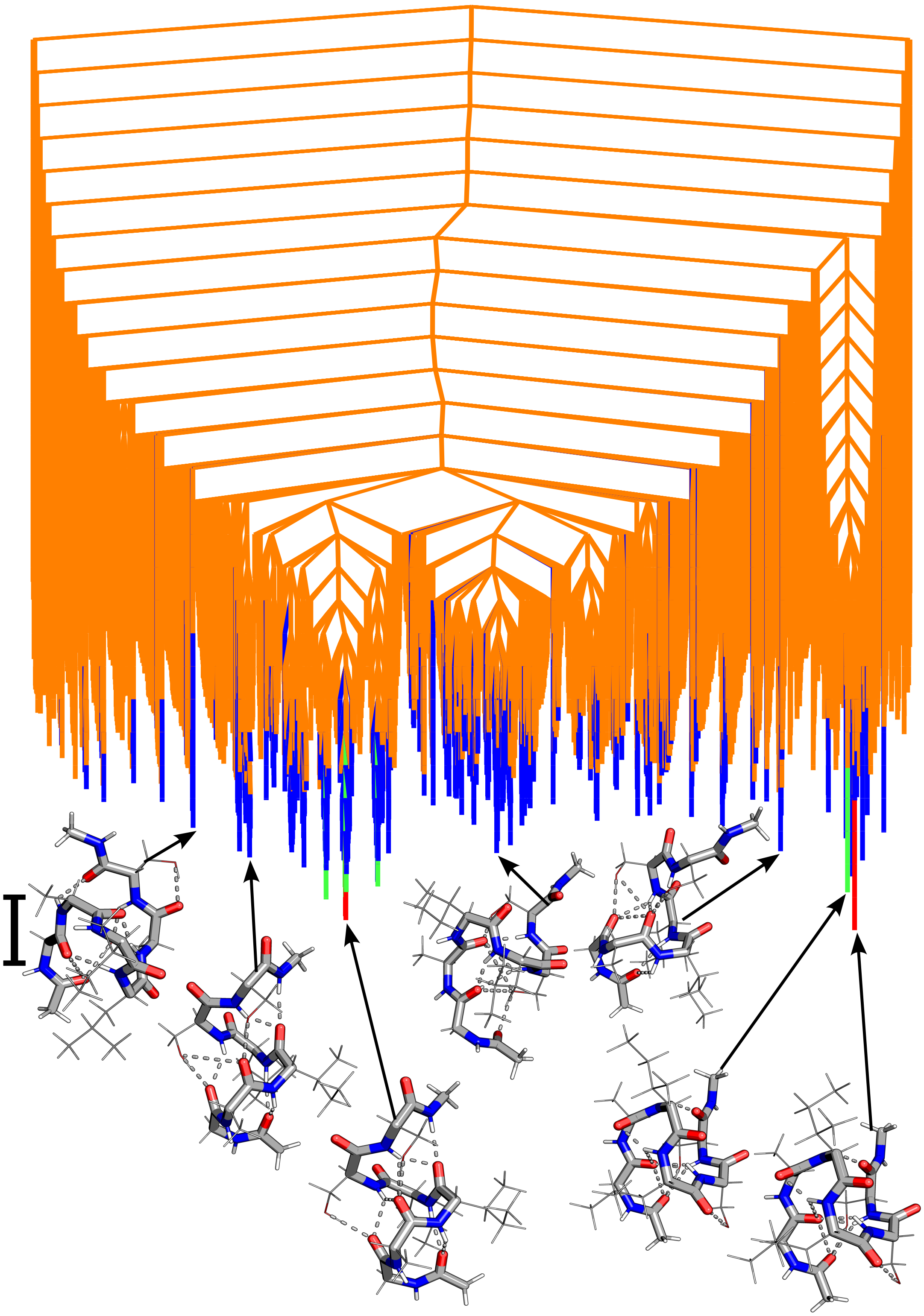

Figure S20: Disconnectivity graphs for **GAILSS** monomer. Local minima representing transition for peaks/inflection points are represented by red to blue (peak 1), green to orange (peak 2), pink to purple (peak 3) and grey to yellow (peak 4). The scalebar represents 1 kcal mol^{-1}.

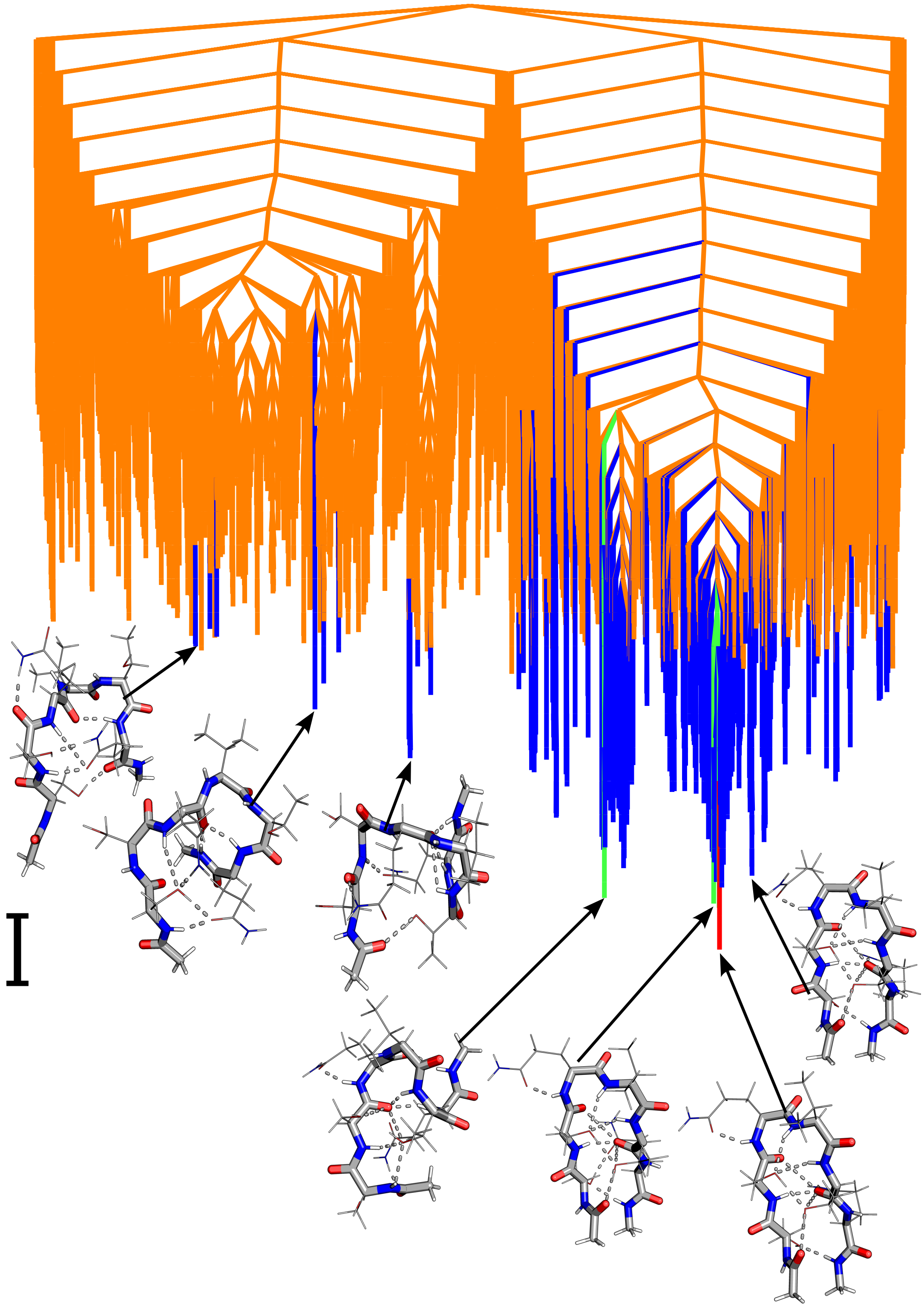

Figure S21: Disconnectivity graphs for **SSQVTQ** monomer. Local minima representing transition for peaks/inflection points are represented by red to blue (peak 1), green to orange (peak 2), pink to purple (peak 3) and grey to yellow (peak 4). The scalebar represents 1 kcal mol^{-1}.

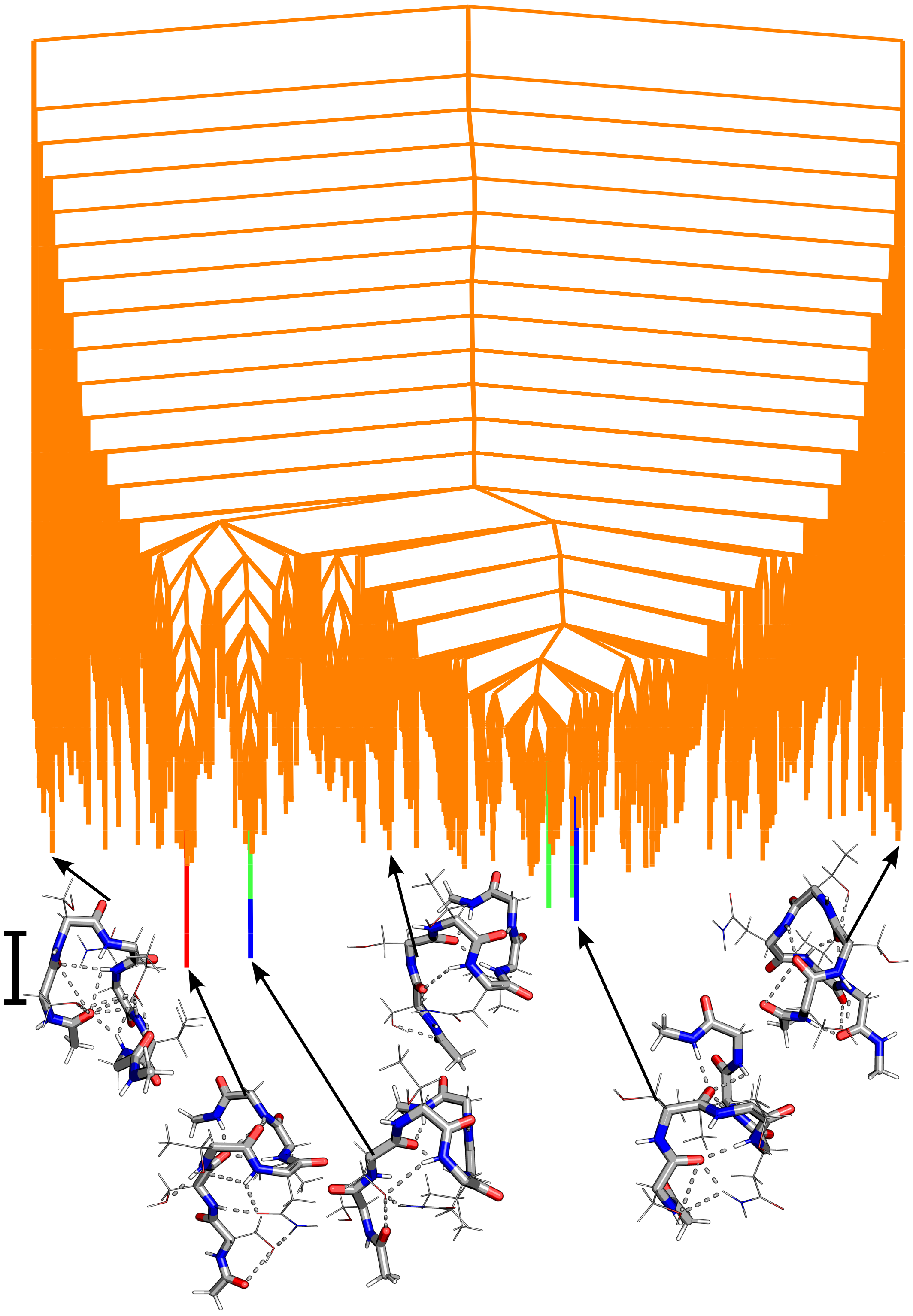

Figure S22: Disconnectivity graphs for **SSTNVG** monomer. Local minima representing transition for peaks/inflection points are represented by red to blue (peak 1), green to orange (peak 2), pink to purple (peak 3) and grey to yellow (peak 4). The scalebar represents 1 kcal mol^{-1}.

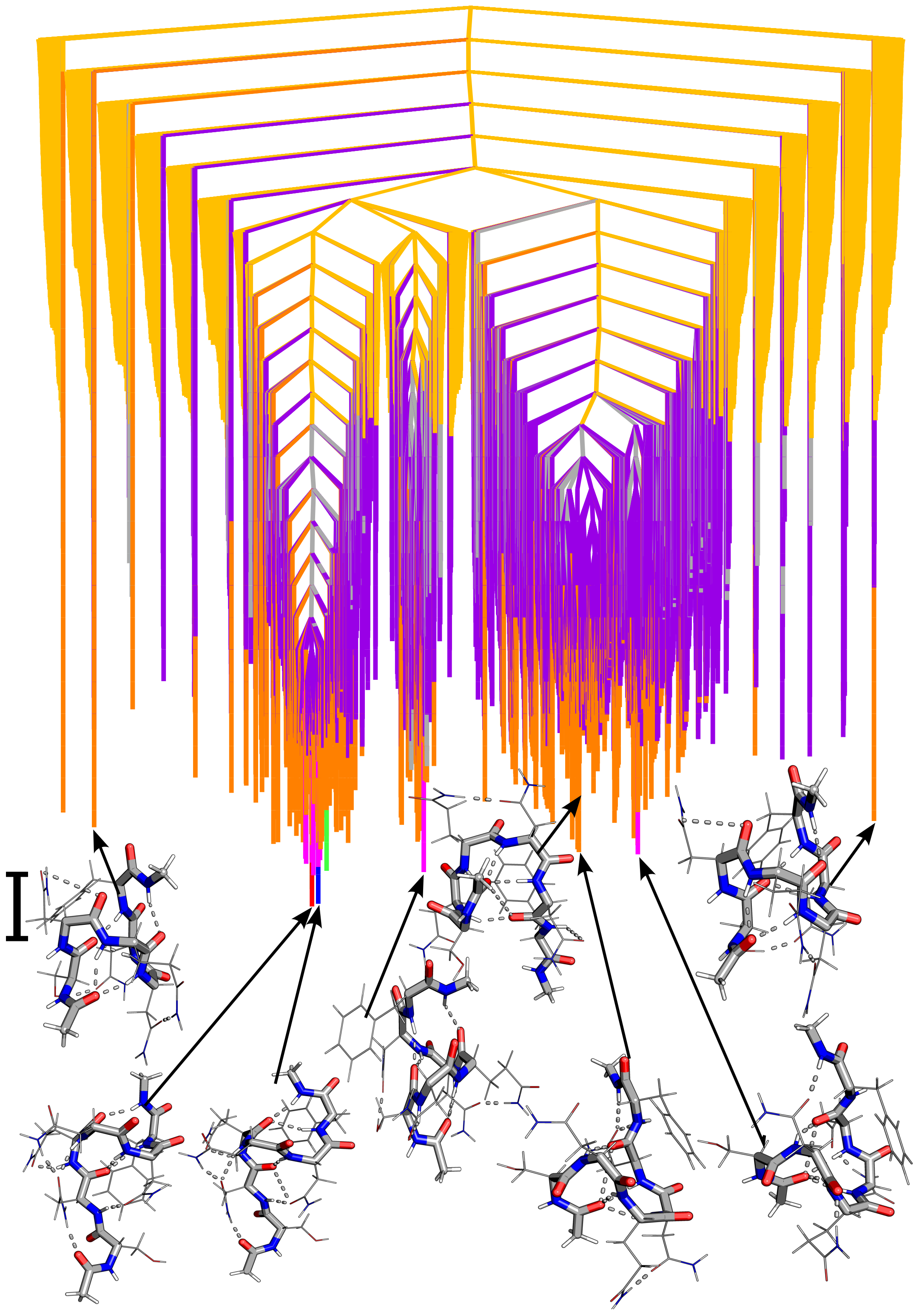

Figure S23: Disconnectivity graphs for **SNQNNF** monomer. Local minima representing transition for peaks/inflection points are represented by red to blue (peak 1), green to orange (peak 2), pink to purple (peak 3) and grey to yellow (peak 4). The scalebar represents 1 kcal mol^{-1}.

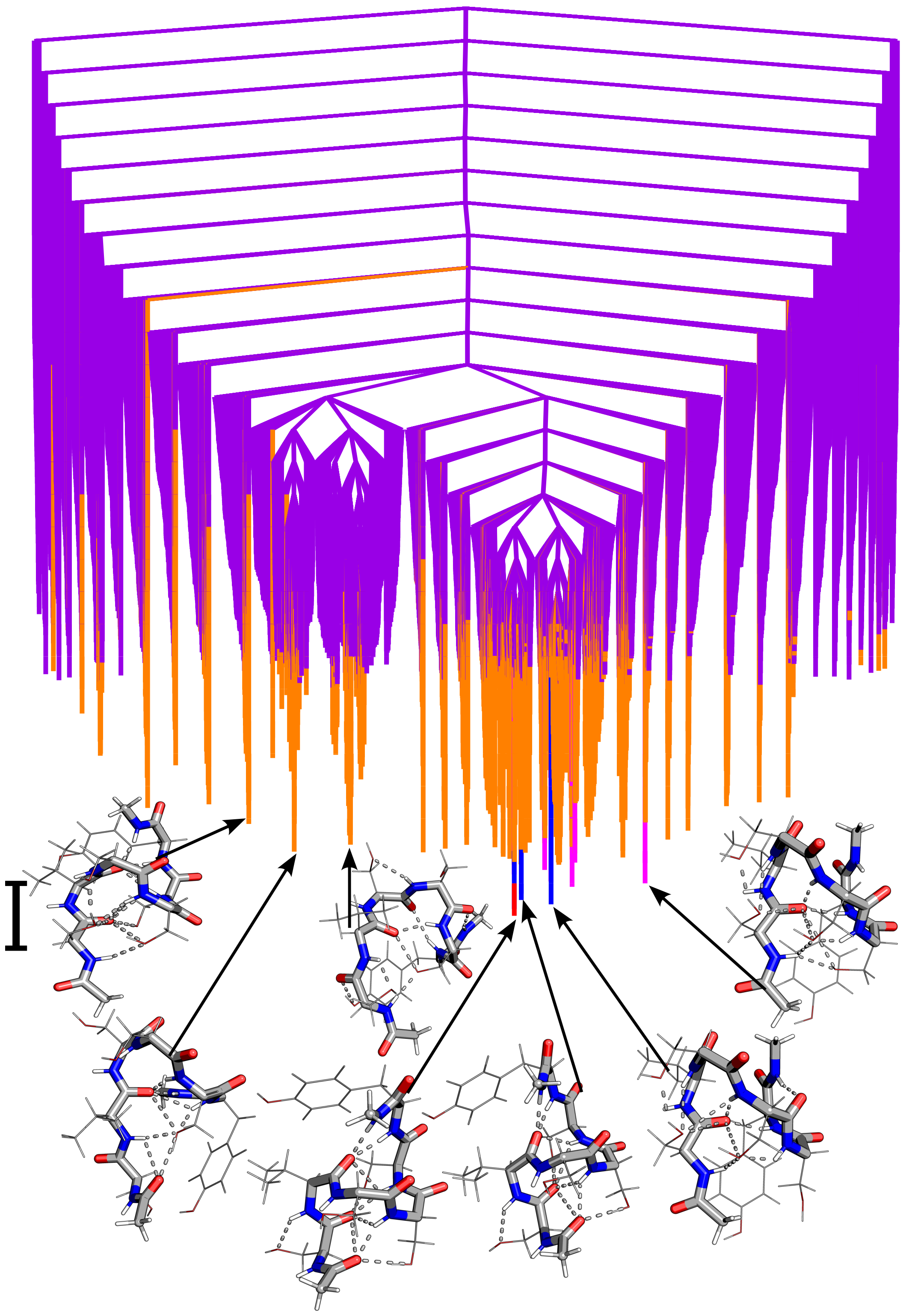

Figure S24: Disconnectivity graphs for **SVSSSY** monomer. Local minima representing transition for peaks/inflection points are represented by red to blue (peak 1), green to orange (peak 2), pink to purple (peak 3) and grey to yellow (peak 4). The scalebar represents 1 kcal mol^{-1}.

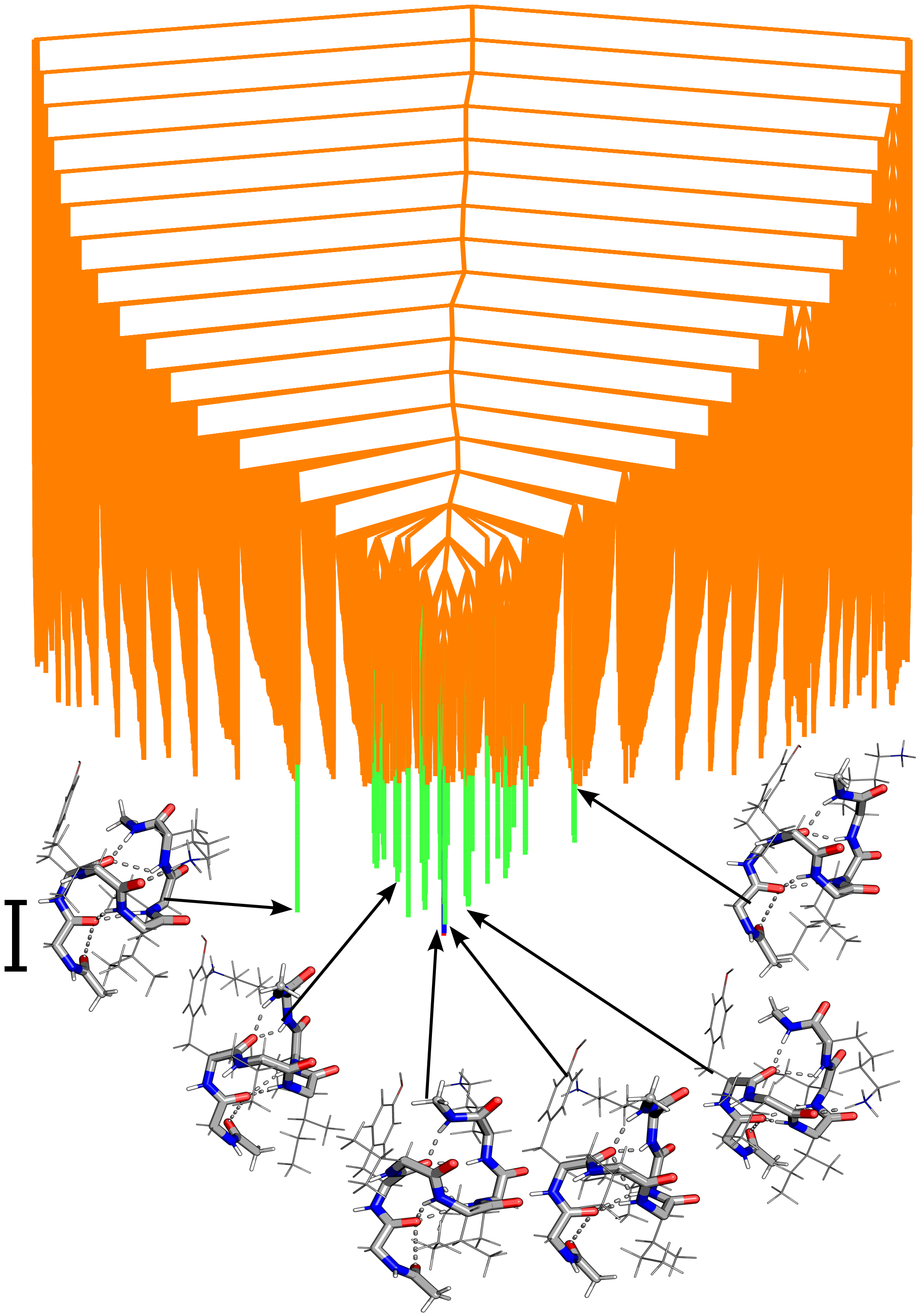

Figure S25: Disconnectivity graphs for **GYVIIK** monomer. Local minima representing transition for peaks/inflection points are represented by red to blue (peak 1), green to orange (peak 2), pink to purple (peak 3) and grey to yellow (peak 4). The scalebar represents 1 kcal mol^{-1}.

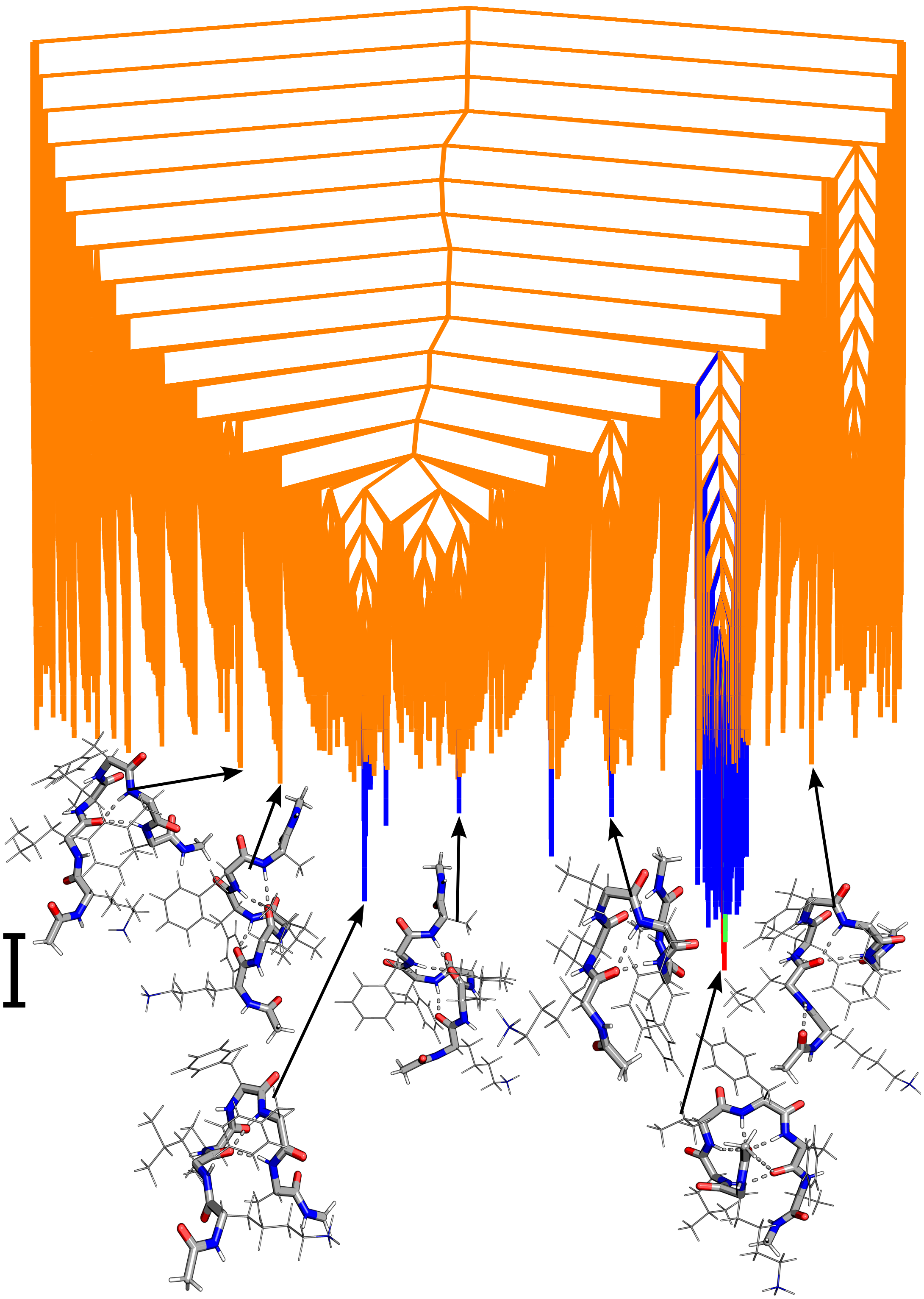

Figure S26: Disconnectivity graphs for **KLVFFA** monomer. Local minima representing transition for peaks/inflection points are represented by red to blue (peak 1), green to orange (peak 2), pink to purple (peak 3) and grey to yellow (peak 4). The scalebar represents 1 kcal mol^{-1}.

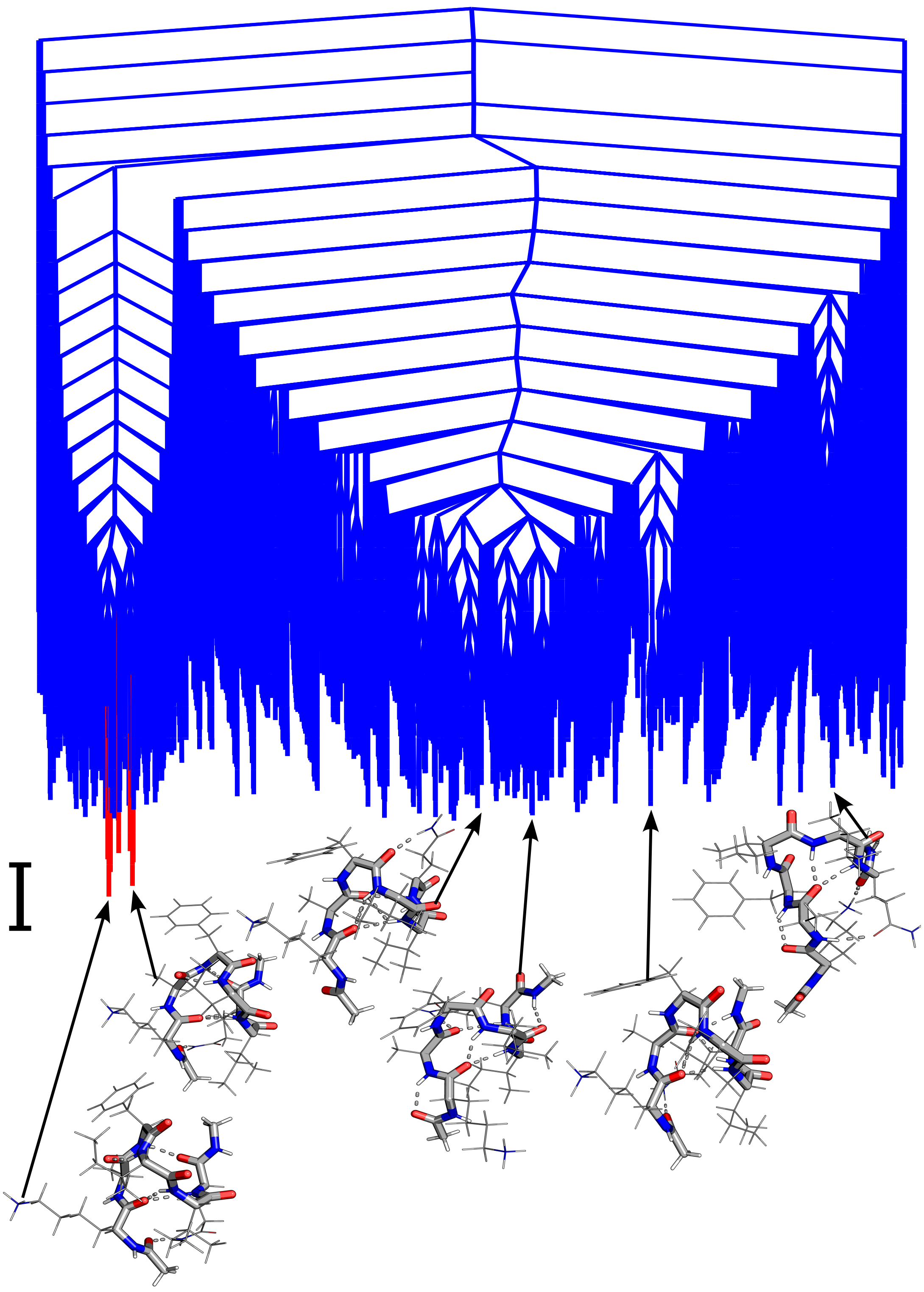

Figure S27: Disconnectivity graphs for KAFIIQ monomer. Local minima representing transition for peaks/inflection points are represented by red to blue (peak 1), green to orange (peak 2), pink to purple (peak 3) and grey to yellow (peak 4). The scalebar represents 1 kcal mol^{-1}.

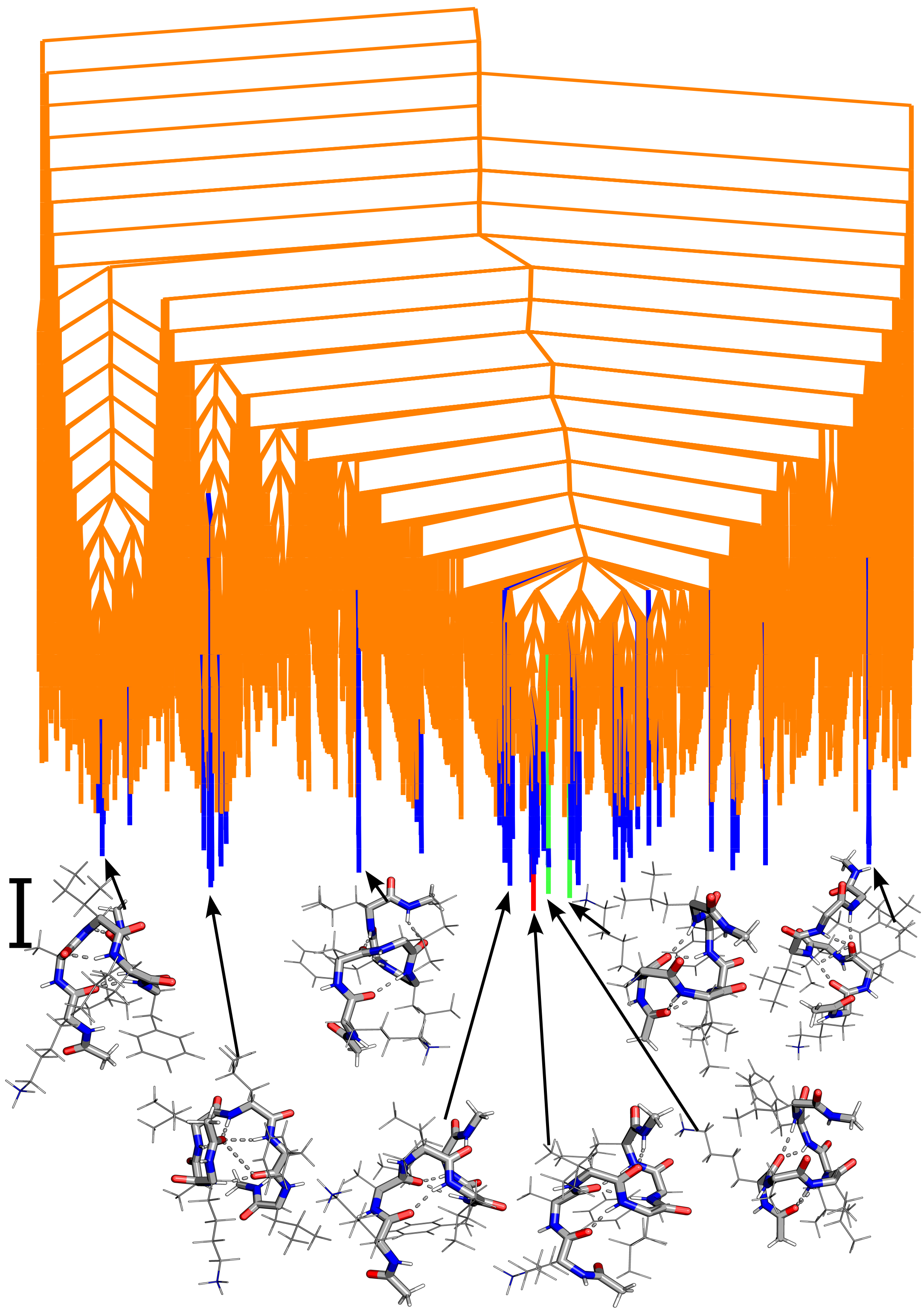

Figure S28: Disconnectivity graphs for KAILFL monomer. Local minima representing transition for peaks/inflection points are represented by red to blue (peak 1), green to orange (peak 2), pink to purple (peak 3) and grey to yellow (peak 4). The scalebar represents 1 kcal mol^{-1}.

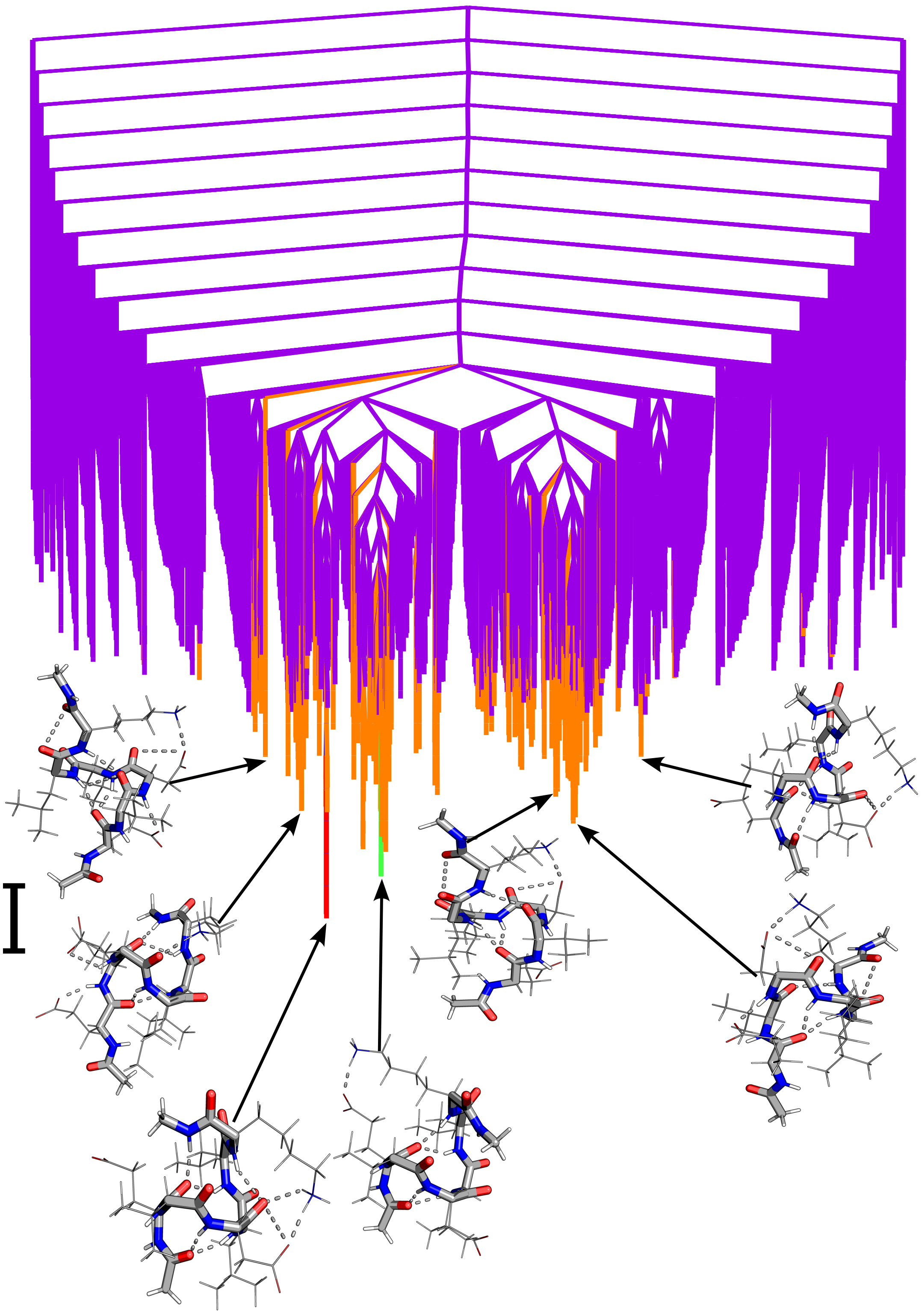

Figure S29: Disconnectivity graphs for EVDLLK monomer. Local minima representing transition for peaks/inflection points are represented by red to blue (peak 1), green to orange (peak 2), pink to purple (peak 3) and grey to yellow (peak 4). The scalebar represents 1 kcal mol^{-1}.

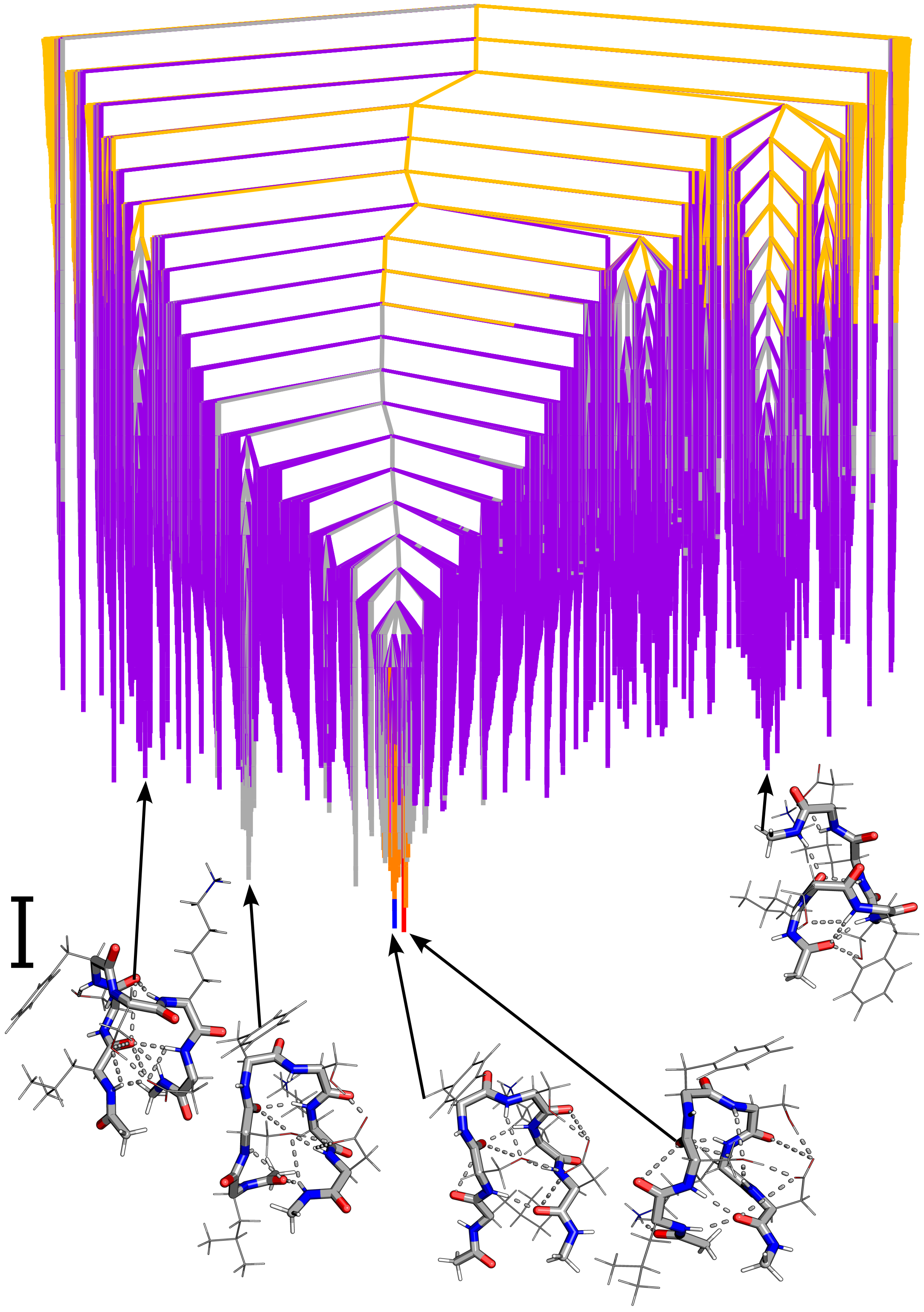

Figure S30: Disconnectivity graphs for LSFSKD monomer. Local minima representing transition for peaks/inflection points are represented by red to blue (peak 1), green to orange (peak 2), pink to purple (peak 3) and grey to yellow (peak 4). The scalebar represents 1 kcal mol^{-1}.

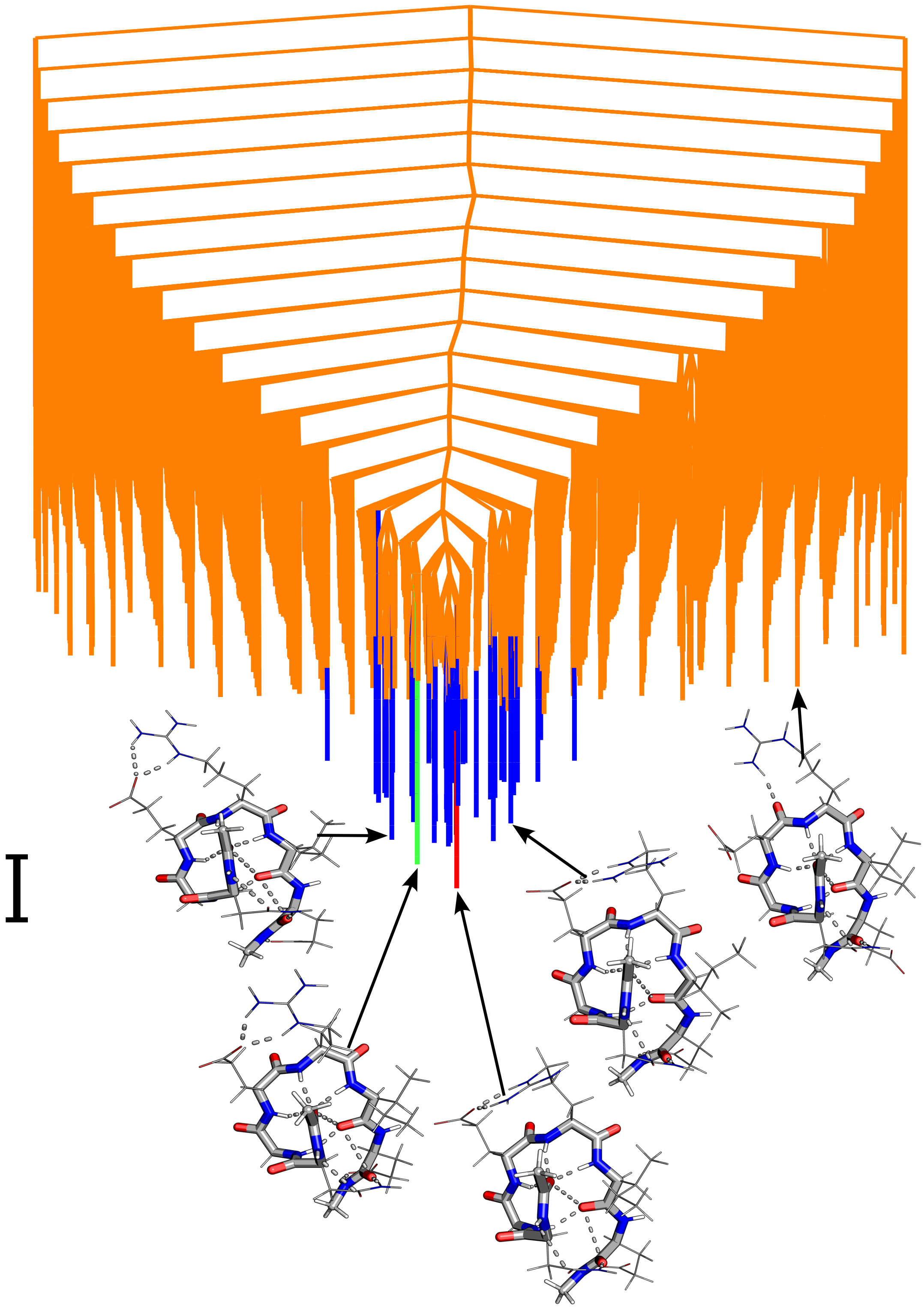

Figure S31: Disconnectivity graphs for NGERIE monomer. Local minima representing transition for peaks/inflection points are represented by red to blue (peak 1), green to orange (peak 2), pink to purple (peak 3) and grey to yellow (peak 4). The scalebar represents 1 kcal mol^{-1}.
